# VEGFR2 deletion increases susceptibility to photoreceptor degeneration through glial-neuronal interaction

**DOI:** 10.1038/s41419-026-08963-z

**Published:** 2026-06-11

**Authors:** Christina B. Bielmeier, Sabrina I. Schmitt, Verena Lehr, Anita Grundl, Andrea E. Dillinger, Herbert Jägle, Christine von Toerne, Stefanie M. Hauck, Süleyman Ergün, Ernst R. Tamm, Anja Schlecht, Andreas Neueder, Barbara M. Braunger

**Affiliations:** 1 Institute of Neuroanatomy, University Medical Center Hamburg-Eppendorf (UKE), Hamburg, Germany; 2 Institute of Human Anatomy and Embryology, University of Regensburg, Regensburg, Germany; 3 Institute of Anatomy and Cell Biology, Julius-Maximilians-University, Wuerzburg, Germany; 4 Department of Ophthalmology, University of Regensburg, Regensburg, Germany; 5 Metabolomics and Proteomics Core, Helmholtz Zentrum München, German Research Center for Environmental Health, Munich, Germany; 6 Atlas University Research Center (ARC), Istanbul Atlas University, Istanbul, Turkey; 7 Institute for Molecular Neurogenetics, Center for Molecular Neurobiology (ZMNH), University Medical Center Hamburg-Eppendorf (UKE), Hamburg, Germany

**Keywords:** Translational research, Neurovascular disorders

## Abstract

Intraocular anti-VEGFA injections are frequently used to counteract neovascularization in diseases such as the neovascular form of age-related macular degeneration (AMD). However, in the clinical context, patients with atrophic (dry) and neovascular (wet) AMD in the same eye will receive anti-VEGFA injections, but there are reports that this severely promotes the development of atrophic areas in dry AMD. To study this, we used mice with a tamoxifen-dependent deletion of Vegfr2 in the eye (*Vegfr2*^Δ*eye*^) and the light damage model to mimic certain aspects of dry AMD. We examined retinal morphology and function, the degree of photoreceptor degeneration, and alterations of the retinal proteome. While steady-state retinal morphology and function were not altered due to VEGF signaling deficiency, light-induced photoreceptor degeneration was drastically exacerbated in *Vegfr2*^Δ*eye*^ retinae, concomitant with attenuated activation of the AKT kinase pathway. Furthermore, using single nuclei RNA sequencing data, we showed that in humans, *Vegfr2* is predominantly expressed in macroglial cells of healthy, dry- and wet AMD retinae. This suggests a VEGF-dependent neuroprotective crosstalk from macroglial cells to photoreceptors and poses promising therapeutic options to attenuate photoreceptor degeneration in humans. Yet, our data also indicate that anti-VEGF therapy should be carefully considered in the presence of neurodegenerative comorbidities.

## Introduction

Age-related macular degeneration (AMD) is among the primary causes of blindness in the elderly population in Western countries [1–3]. Intriguingly, with progression of the disease, AMD potentially develops into a neovascular form, which is characterized by the formation of new vessels [4]. These neovascularizations typically arise in the posterior part of the eye; in the case of neovascular (wet) AMD, blood vessels from the choriocapillaris invade the subretinal space [5, 6]. The pathogenesis of neovascular AMD is characterized by endothelial proliferation and migration, an increased vascular permeability and inflammation, finally resulting in retinal neurodegeneration [7]. Intriguingly, vascular endothelial growth factor (VEGF) signaling plays a key role in these processes. This observation led to the development and use of anti-VEGF therapies, such as blocking antibodies or receptor traps, to counteract these alterations [8].

Canonical VEGF signaling is activated through receptor binding of specific ligands (e.g., VEGFA-D and placental growth factor (PIGF)), with VEGFA being the best described and characterized factor [9, 10]. In the context of the vascular and nervous system, VEGF signaling is mainly mediated through specific binding of the ligands to VEGF receptor 1 (VEGFR1 or FLT-1), VEGFR2 (KDR or FLK-1) and their co-receptor neuropilin-1 (NRP-1), respectively [9, 11]. Even though VEGFA binds VEGFR1 with higher affinity than VEGFR2, the VEGFR1-mediated kinase activity is much weaker compared to the activity of VEGFR2 [12–14]. Therefore, VEGFR2 is considered to be the main receptor for VEGF-mediated signaling [9].

Late stages of AMD may also appear as geographic atrophies (dry AMD), which are characterized by atrophic lesions of the outer retina and loss of the retinal pigment epithelium (RPE), leading to progressive and irreversible loss of the central vision [4, 6, 15]. Of note, both states, dry and neovascular (wet) AMD, can coexist in the same eye [16], and there is emerging evidence that treatment of the neovascular form with anti-VEGF may increase the likelihood of developing or promoting the progression of geographic atrophies in dry AMD [17].

In this study, we show that deletion of VEGF signaling increases susceptibility of photoreceptors towards degeneration in a mouse model mimicking dry AMD [18–20]. We furthermore show that VEGF signaling acts neuroprotectively for photoreceptor survival through modulation of the neuroprotective AKT (protein kinase B) signaling pathway. Single nuclei sequencing of healthy and diseased (dry and wet AMD) human retinae furthermore suggests a VEGF-signaling-dependent crosstalk from glial cells to photoreceptors promoting neuroprotection. Yet, deletion of VEGF signaling in the otherwise healthy retina does not obviously affect its morphology, function, or proteome.

Our findings are of great clinical importance as it has been hypothesized that VEGF acts as a survival factor for photoreceptors [21]. Our data furthermore provide experimental evidence regarding clinical observations that in patients with dry and wet AMD in the same eye, the atrophic areas increase after anti-VEGF therapy [17].

## Material and methods

### Mice

All procedures conformed to the tenets of the National Institutes of Health Guidelines on the Care and Use of Animals in Research, the EU Directive 2010/63/E and institutional guidelines. *Vegfr2*^*fl/fl*^ [22] and tamoxifen-dependent *CAGGCre-ER*^*TM*^ [23] that were hemizygous for Cre were crossbred, resulting in *CAGGCre-ER*^*TM*^*;Vegfr2*^*fl/fl*^ or *Vegfr2*^*fl/fl*^ offspring. All mice were albino (CD1/FVB-N genetic background), negative for RD1 and homozygous for the light-sensitive L450 variant of retinal pigment epithelium-specific protein 65 kDa (RPE65, L450 variant, with leucine at position 450 of RPE65) [24]. Translocation of Cre in the nucleus was achieved by application of 10 µl tamoxifen-containing eye drops (5 mg/ml, Cayman Chemicals, Ann Arbor, Michigan, USA) in corn oil (Sigma-Aldrich, Taufkirchen, Germany)) in 1-month-old animals. Eye drops were given 3 times per day at intervals of 4 h for 5 consecutive days.

For simplicity, tamoxifen-treated *CAGGCre-ER*^*TM*^*;Vegfr2*^*fl/fl*^ mice will be referred to as *Vegfr2*^Δ*eye*^ and tamoxifen-treated *Vegfr2*^*fl/fl*^ littermates, representing functional wildtype animals, will be referred to as wildtype. All experiments were performed in mice of either sex. The approximate sample size was determined a priori using a statistical power analysis. To avoid intentional bias in the selection of the individual animals, assignment to the experimental groups (*Vegfr2*^Δ*eye*^, wildtype, dark adaptation, light exposure) was performed using a random assignment procedure. This approach was applied uniformly to all samples, and investigators were blinded to both genotype and treatment when analyzing the data.

### Genotyping

All mice were screened by isolating genomic DNA from tail/ear biopsies and tested by PCR for *Cre* and *Vegfr2* using the primer pairs that are listed in Table 1 and protocols as previously described [25]. Briefly, for *Cre* and *Vegfr2* genotyping, the thermal cycle profile was denaturation at 96 °C for 30 s, annealing at 58 °C for 30 s, and extension at 72 °C for 1 min for 35 cycles. L450 variant of RPE65 was confirmed by PCR as detailed in [26].

**Table 1:** Primer sequences for genotyping, *wt* wildtype, *flox* floxed allele.

Gene	Sequence	Product size
*Cre*	5’-atgcttctgtccgtttgccg-3’ 5’-cctgttttgcacgttcaccg-3’	270 bp
*Vegfr2*	5’- ccacagaacaactcagggcta-3’ 5’- gggagcaaagtctctggaaa-3’	wt: 179 bp flox: 230 bp
*RPE65*	5’-cactgtggtctctgctatcttc-3’ 5’-ggtgccagttccacttcagtt-3’	methionin: 674 bp leucin: 437 + 236 bp

### Light microscopy

Mice were sacrificed, and the eyes were carefully enucleated and fixed for 24 h in Ito’s fixative [27]. The eyes were marked with a thin, short metal needle at the superior limbus and embedded in Epon (Serva, Heidelberg, Germany). 1.0 μm thick semithin sections were cut along the mid-horizontal plane (in nasal-temporal orientation), stretching through the optic nerve head (ONH) and the pupil. Sections were stained according to Richardson’s protocol [28] and images taken using an Axio Imager Z1 light/fluorescent microscope (Carl Zeiss, Göttingen, Germany). The thickness of the outer nuclear layer (ONL) was measured at nine equidistant loci along the circumference of each hemisphere as described in [29, 30]. The means and corresponding standard errors of the mean (SEM) were calculated for each measurement point, and the results were plotted as a spider diagram.

### Immunohistochemistry and dextran perfusion

Enucleated eyes were fixed for 4 h (sections) or 2 h (retinal flatmounts) in 4% paraformaldehyde (PFA) at room temperature (RT), and then washed extensively in phosphate buffer (PB, 0.1 M, pH 7.4). Eyes for plasmalemmal vesicle-associated protein-1 (PLVAP/PV1) immunohistochemistry were embedded in paraffin. Paraffin sections (6 μm) were deparaffinized, rehydrated in H_2_O and stained for PV1 (Santa Cruz Biotechnology, sc-19603) as previously described.

For VEGFR2 staining, deparaffinized sections underwent antigen retrieval in 10 mM Tris–EDTA buffer (pH 9.0) at boiling temperature for 15 min, followed by blocking in 2% BSA and 0.1% Triton X-100 in PB (0.1 M) for 1 h at RT. Sections were incubated overnight at 4 °C with rabbit anti-VEGFR2 primary antibody (Cell Signaling Technology, clone 55B11; 1:2000 in blocking solution (1:10)). After three washes in PB (5 min each), sections were incubated with goat anti-rabbit Alexa Fluor 647 secondary antibody (Invitrogen, A-21246; 1:1000 in blocking solution (1:10)) for 1 h at RT. Glutamine synthetase (GS; Merck, MB302; 1:100 in blocking solution (1:10)) immunofluorescence staining was performed using a chicken anti-mouse Alexa Fluor 488 secondary antibody (Invitrogen, A-21200; 1:500 in blocking solution (1:10)) according to previously published protocols [31, 32]. Sections were washed three times in PB, and nuclei were counterstained with DAPI (20 µg/ml; Sigma-Aldrich) for 5 min. Finally, sections were mounted using fluorescence mounting medium (Dako, Agilent Technologies).

High-molecular-weight FITC-dextran (MW = 2,000,000 g/mol; Sigma-Aldrich) perfusions were performed on deeply anaesthetized animals. Retinal flatmounts were prepared from 4% PFA-fixed eyes as previously described. For FITC-dextran-perfused sagittal sections, the eyes were incubated in 10%, 20%, and 30% sucrose/PBS overnight at 4° C, and shock-frozen in tissue mounting medium (DiaTec). Following sectioning, nuclei were counterstained with DAPI (Vectashield, Vector Laboratories) at a dilution of 1:10 in fluorescence mounting medium (Serva). Retinal sections and flatmounts were imaged using an Axio Imager Z1 fluorescence microscope (Carl Zeiss) under identical acquisition settings.

### VEGFR2 fluorescence intensity quantification

Fluorescence intensity was quantified using ImageJ (NIH, USA). Regions of interest (ROIs) were manually defined (choroid and retina or neural retina). For each ROI, the integrated density and area were measured. To account for differences in ROI size, fluorescence intensity was normalized to the respective area, yielding normalized fluorescence intensity values expressed in arbitrary units [AU]. All images were acquired using identical microscope settings (including exposure time and gain) and processed on the same day to ensure comparability between samples.

### Functional electroretinogram (ERG) analyses and in vivo imaging

Mice were dark-adapted for at least 12 h and were anesthetized by subcutaneous injection of ketamine (65 mg/kg) and xylazine (13 mg/kg) right before the experiments. Pupils were dilated with tropicamide eye drops (Mydriaticum Stulln; Pharma Stulln). Silver needle electrodes served as a reference (forehead) and a ground (tail), and gold wire ring electrodes served as active electrodes. Corneregel (Bausch & Lomb) was applied to keep the eye hydrated and to maintain good electrical contact. ERGs were recorded using a Ganzfeld bowl (Ganzfeld QC450 SCX; Roland Consult) and an amplifier and recording unit (RETI-Port; Roland Consult). ERGs were recorded from both eyes simultaneously, bandpass filtered (1–300 Hz), and averaged. Single flash scotopic (dark-adapted) responses to a series of 10 LED-flash intensities ranging from −3.5 to 1.0 log cd.s/m^2^ with an interstimulus interval of 2–20 s for the highest intensity were recorded. After 10 min of adaptation to white background illumination (25 cd/m^2^), single flash photopic (light-adapted) responses to three Xenon-flash intensities (1, 1.5, and 2 log cd.s/m^2^). All analysis and plotting were performed with R 4.4.2 (The R Foundation for Statistical Computing) and ggplot2 3.5.2 [33].

### Fundus imaging and angiography

Retinal imaging was performed with a commercially available imaging system (Micron III; Phoenix Research Labs, Pleasanton, CA, USA). Light source path and imaging path filters (low pass and high pass at 500 nm) were used for fluorescein angiography (FLA). Mice were anesthetized, and pupils dilated as described before. FLA was performed using a subcutaneous injection of 75 mg/kg body weight fluorescein-Na (ALCON Pharma GmbH, Freiburg, Germany).

### Sample preparation for mass spectrometry (MS)

Both retinae of an animal were thawn by adding ice-cold 200 µl tris buffered saline (TBS) containing 1% Nonidet P (NP)-40 with protease inhibitors (complete^TM^, Roche). The mixture was then transferred to a Precellys-Tube for homogenization using the Precellys Tissue Homogenizer tubes filled with small balls and 2 big ceramic beads (Soft tissue homogenizing CK14, – 0,2 mL; Bertin, Montigny-le-Bretonneux, FR) for tissue disruption. The samples were homogenized by shaking twice for 20 s at 5500 cycles per minute. Lysates were centrifuged for 10 min at 10,000 × *g* and supernatant transferred into a fresh tube. Protein content was determined by Bradford assay (Biorad, Hercules, CA, USA). Equal amounts of tissue lysates were subjected to tryptic digest, applying a modified filter-aided sample preparation (FASP) procedure as described in [34]. Peptides were collected by centrifugation (10 min at 14,000 *g*), acidified with trifluoroacetic acid (TFA), and stored at –20 °C.

The MS data were acquired in DIA mode on a Q Exactive HF mass spectrometer (Thermo Fisher Scientific Inc., Waltham, MA, USA). Equal amounts of peptides were automatically loaded to the online coupled RSLC (Ultimate 3000, Thermo Fisher Scientific Inc.) HPLC system. A Nano-Trap column was used (300-µm inner diameter (ID) × 5 mm, packed with Acclaim PepMap100 C18, 5 µm, 100 Å from LC Packings, Sunnyvale, CA, USA) before separation by reversed-phase chromatography (Acquity UPLC M-Class HSS T3 Column 75 µm ID × 250 mm, 1.8 µm from Waters, Eschborn, Germany) at 40 °C. Peptides were eluted from the column at 250 nl/min using increasing ACN concentration in 0.1% formic acid from 3 to 40% over a 90-min gradient. The DIA method consisted of a survey scan from 300 to 1650 m/z at 120000 resolution and an automatic gain control (AGC) target of 3e6 or 100 ms maximum injection time. Fragmentation was performed via higher-energy collisional dissociation with a target value of 3e6 ions determined with predictive AGC. Precursor peptides were isolated with 37 variable windows spanning from 300 to 1650 m/z at 30000 resolution with an AGC target of 3e6 and automatic injection time. The normalized collision energy was 28, and the spectra were recorded in profile type. The mass spectrometry proteomics data have been deposited to the ProteomeXchange Consortium via the PRIDE [35] partner repository with the dataset identifier PXD066263.

### Data analysis of MS spectra

DIA files were processed with Spectronaut (Version 15, Biognosys) as direct DIA analysis against a SwissProt mouse database (17081 sequences; release 2020_02 including spike proteins), using BSG factory settings for Pulsar with variable modifications set to acetylation of protein N-terminus, deamidation (NQ) and Oxidation (M). For DIA analysis, default settings were applied with the following changes: For quantification, precursor filtering was set on Qvalue and the proteotypicity filter was set on only proteotypic. The LFQ method was set to Quant 2.0 algorithm, quantity MS level was MS2, quantity type was area, cross-run normalization was set to local normalization and major group quantity was calculated as mean TOP3 peptide quantity. Data were exported without cross-run normalization for further analysis.

All subsequent analyses were conducted in R v4.2.1. After removal of non-unique identifiers, the raw protein LFQ intensities of 3809 proteins were normalized using a variance stabilizing normalization (vsn) [36]. We used Euclidean distance and principal component analysis to analyze sample relationships and defined one outlier sample, which was removed from the following analysis. This left *n* = 6 for both light-damage groups and *n* = 5 for both dark-adapted groups. Next, we corrected for the sex of the animals using limma [37]. Dysregulation was computed using DEqMS [38] with a contrast table and the number of peptides per protein as a coefficient during model fitting of the vsn-normalized, sex-corrected LFQ intensities. DEqMS analysis resulted in a total of 3729 proteins, as some proteins were removed during the analysis due to too many missing data points per group.

### Light damage (LD) experiments

The mice were reared in 12 h light/12 h dark cycles (lights on at 7 a.m., light intensity approx. 400 lux). Five days before light exposure, six-week-old animals were dark-adapted in cyclic dim light ( < 50 lux) followed by a period of complete darkness for 18 h. Light damage (LD) experiments were always performed in the early morning with an intensity of 5000 lux cool white light for 30 min as described in [29]. Light damage experiments were conducted with one mouse per cage. Up to four cages were exposed to light simultaneously. Therefore, the light damage experiments were repeated at least 26 times.

Animals were sacrificed at the following time points after light exposure: 6 h (for RNA, proteome and western blot analysis), 30 h (for Terminal deoxynucleotidyl transferase-mediated dUTP nick end labeling (TUNEL)) or 14 days (for morphometric analyses). Dark-adapted (DA) animals (*Vegfr2*^Δ^^eye^ and wildtype littermates) that were not treated with the light-damage paradigm were used as reference groups for the light-damage paradigm.

### TUNEL labeling and quantification

TdT-mediated dUTP-biotin nick end labeling (TUNEL, Promega, Madison, Wisconsin, USA) was conducted on 4% PFA (in 0.1 M PP, pH 7.4) fixed and paraffin-embedded eyes according to previously published protocols [30]. TUNEL labeling was performed in five independent experiments. Stained sections were visualized by fluorescence microscopy using the Axio Imager Z1 (Carl Zeiss, Göttingen, Germany). The number of TUNEL-positive nuclei per ONL was counted using the software ImageJ and normalized to mm^2^ ONL area as described in [29].

### RNA analysis

Total RNA was extracted from neural retinae using TriFast (VWR/Avantor, Radnor, Pennsylvania, USA), and first-strand cDNA synthesis was performed using the iScript cDNA Synthesis Kit (Bio-Rad Laboratories, Inc., Hercules, California, USA) according to the manufacturer’s instructions. A CFX Realtime PCR Detection System was used for quantitative RT-PCR (qPCR) analyses. The temperature profile was denaturation at 95 °C for 10 s and annealing and extension at 60 °C for 40 s for 40 cycles. RNA that was not previously reverse transcribed and H_2_O served as negative controls. All primer pairs (for sequences please see Table 2) were purchased from Invitrogen/Thermo Fisher Scientific (Carlsbad, California, USA) and designed to extend over exon–intron boundaries. CFX Manager^TM^ Software and Excel (Microsoft Corporation, Redmond, WA, USA) were used to analyse relative mRNA expression levels according to the ∆∆C_T_-method [39]. The mean values of the reference genes guanine nucleotide binding protein subunit beta2-like 1 *(Gnb2l1)*, ribosomal protein L32 *(Rpl32)* and glyceraldehyde 3-phosphate dehydrogenase *(Gapdh)*/ubiquitin c (*Ubc*, for *Vegfa* qPCR), were used for normalization.

**Table 2:** Primers for quantitative real-time RT-PCR.

Gene	Sequence fwd.	Sequence rev.
*Gapdh*	5’-tgtccgtcgtggatctgac-3’	5’-cctgcttcaccaccttcttg-3’
*Gnb2l1*	5’-tctgcaagtacacggtccag-3’	5’-acgatgatagggttgctgct-3’
*Rpl32*	5’-gctgccatctgttttacgg-3’	5’-gactggtgcctgatgaact-3’
*Ubc*	5‘-gtctgctgtgtgaggactgc-3‘	5‘-cctccagggtgatggtctta-3‘
*Vegfa120*	5‘-aacgatgaagccctggagtg-3‘	5‘-tgagaggtctggttcccga-3‘
*Vegfa164*	5‘-aacgatgaagccctggagtg-3‘	5‘-gacaaacaaatgctttctccg-3‘
*Edn2*	5’-acctcctccgaaagctgag-3’	5’-tttcttgtcacctctggctgta-3’
*Lif*	5’-aaacggcctgcatctaagg-3’	5’-agcagcagtaagggcacaat-3’
*Fgf2*	5’-cggctctactgcaagaacg-3’	5’-tgcttggagttgtagtttgacg-3’
*Gfap*	5′-acagactttctccaacctccag-3′	5′-ccttctgacacggatttggt-3′
*Vegfr2*	5′-aaatacaacccttcagattacttgc-3′	5′-cagaatcacgctgagcatt-3′

### Western blot analyses

Retinal proteins were isolated following the manufacturer’s instructions (Invitrogen) for TRIzol protein isolation. Proteins were separated by SDS-PAGE (10% gel electrophoresis) and transferred by semidry blotting onto activated polyvinyl difluoride membranes (PVDF; Millipore, Burlington, Massachusetts, USA), which were incubated with blocking reagent (Table 3) in Tris-buffered saline (TBS) containing 0.1% Tween 20 (TBS-T, pH 7,2) and then incubated with primary antibodies as specified in Table 3 overnight. After washing with TBS-T, secondary antibodies were added. Chemiluminescence was detected on a LAS 3000 imaging workstation (Fujifilm, Minato, Tokyo, Japan). For normalization, blots were stained with an antibody for the reference protein glyceraldehyde 3-phosphate dehydrogenase (GAPDH). For uncropped western blot membranes see supplementary material ”unedited gels”. Western blots were evaluated by relative densitometry using the Aida Image Analyzer v.4.06 software (Raytest IDA, Herolabs GmbH, Wiesloch, Germany) and Excel (Microsoft Corporation, Redmond, WA, USA).

**Table 3:** Antibodies and respective blocking reagents used for Western blot analyses.

Primary antibody	Blocking reagent	Secondary antibody
pAKT (Cell Signaling, #4060S); 1:500 in 0.3% BSA in TBS-T	3% BSA in TBS-T for 1 h	Chicken anti-mouse coupled to alkaline phosphatase (Santa Cruz Biotechnology, #sc-2966); 1:2000
AKT (Cell Signaling, #9272S); 1:1000 in 0.5% non-fat dry milk in TBS-T	5% non-fat dry milk in TBS-T for 1 h	Goat anti-rabbit coupled to horseradish peroxidase (Rockland, 611-1322-0500, #ABIN964978); 1:2000
Hif1α (Cayman Chemical, #10006421); 1:200 in 0.5% non-fat dry milk in TBS-T	5% non-fat dry milk in TBS-T for 1 h	anti-rabbit coupled to horseradish peroxidase (Cell Signaling Technology, #7074); 1:5000
Glyceraldehyde 3-phosphate dehydrogenase (Abcam, ab204732); 1:5000 in 0.5% BSA in TBS-T	5% BSA in TBS-T for 30 min	no secondary antibody required

### Single-nuclei sequencing

Single cell RNA sequencing data of healthy human donors (age: 65 years, male; 90 years, female; 81 years, female), individuals with neovascular AMD (age: 67 years, female; 100 years, female; 93, female) and intermediate dry (age: 82 years, female; 72 years, male; 74 years, female) AMD, was obtained from the ENA archive with accession number PRJNA912653 (https://www.ebi.ac.uk/ena/browser/view/PRJNA912653). Reads were mapped and quantified using the Alevin-Fry framework (alevin-fry v0.8.1, salmon v1.10.1, pyroe v0.9.2, python v3.9.16) [40]. To this end, we created a splici index using the Ensembl GRCh38 primary assembly with a read length of 91. Reads were mapped with the salmon alevin command using IU as the library type and ChromiumV3 as the library type. We used sketch mode to generate a rad file by pseudoalignment with structural constraints. Next, we generated a barcode permit list using the 10x Genomics barcodes whitelist (3M-february-2018.txt). Additionally, we filtered reads that mapped to the reverse complement strand of transcripts by specifying -d fw. Lastly, UMIs per-gene and per-cell were quantified using the ‘cr like’ strategy.

All subsequent analyses were conducted in R v4.4.2. For quality control (QC) and filtering, we used the singleCellTK package [41]. After data import from Alevin-Fry, only droplets with more than 100 genes and 500 mapped reads were kept. Next, we filtered droplets by running the runEmptyDrops() and runBarcodeRankDrops() on the single-cell datasets. We removed all droplets with a BarcodeRank_Inflection of 1, all droplets with non-computable FDR and all droplets with FDR < 0.01 for the probability to be an empty droplet. 5 samples (GSM6841144, GSM6841146, GSM6841150, GSM6841151, GSM6841158) had fewer than 300 droplets left and were removed. Next, we ran per-cell QC using the runCellQC() function using the ‘QCMetrics’, ‘scDblFinder’ and ‘decontX’ algorithms. Final droplets were filtered using a mitochondrial percentage of less than 5% and a scDblFinder call of being a ‘singlet’. Droplets with large ambient RNA decontamination ( > 0.5) as determined by decontX were also removed. GSM6841154 and GSM6841155 were removed because both samples had only very low droplet numbers after final QC. Sample GSM6841145 showed a strongly skewed distribution of genes/droplets and was therefore removed, resulting in a final sample number of 3 libraries per genotype.

Final, filtered datasets were imported into Seurat v5.2.1 [42]. To cluster cells, we used Harmony [43] with a dimensionality of 50 for all subsequent analyses. To assign final cell types, we clustered cells with a resolution of 2 and analyzed the expression of cell-type marker genes in each cluster. Clusters expressing the same pattern of marker genes were merged again with guidance of the coordinates of the UMAP dimensions 1 and 2. Marker gene expression in the final clusters is shown in supplementary figure 4.

### Statistics

Data are shown as mean ± SEM (standard error of the mean). The observed variance was comparable between the different groups. Statistical comparative analyses between the mean variables of two individual test populations were performed using a two-tailed Student’s *t* test in Excel (Microsoft Corporation, Redmond, WA, USA). One-way ANOVA analyses were performed in SPSS (IBM Corporation, Armonk, New York, USA) if more than two individual groups were compared (post-hoc test: Bonferroni). *P* values ≤ 0.05 were considered to be statistically significant.

### Ethic approval

Animal experiments conformed to the tenets of the National Institutes of Health Guidelines on the Care and Use of Animals in Research, the EU Directive, 2010/63/E, institutional guidelines and were approved by the Government of Bavaria, Regierung von Unterfranken, Würzburg, Germany (AZ 55.2-2532-2-262). The single-cell sequencing data of human samples is based on the reanalysis of a publicly available dataset. Informed consent was obtained by the original authors as described in the original publication [44].

## Results

### Conditional deletion of *Vegfr2* in the adult eye: retinal morphology and function

Mice with a tamoxifen-dependent conditional deletion of *Vegfr2* (referred to as *Vegfr2*^Δ*eye*^) and their Cre-negative *Vegfr2*^*fl/fl*^ littermates (referred to as wildtype) were treated with tamoxifen-containing eye drops. This resulted in a significant deletion of *Vegfr2* mRNA and VEGFR2 protein (supplementary fig. 1A, ***p = 0.0008; supplementary fig. 1B, **p = 0.002 (retina + choroid); **p = 0.007 (neural retina); for detailed numbers see Table 4; supplementary fig. 1C) in *Vegfr2*^Δ*eye*^ retinae.

**Fig. 1:**
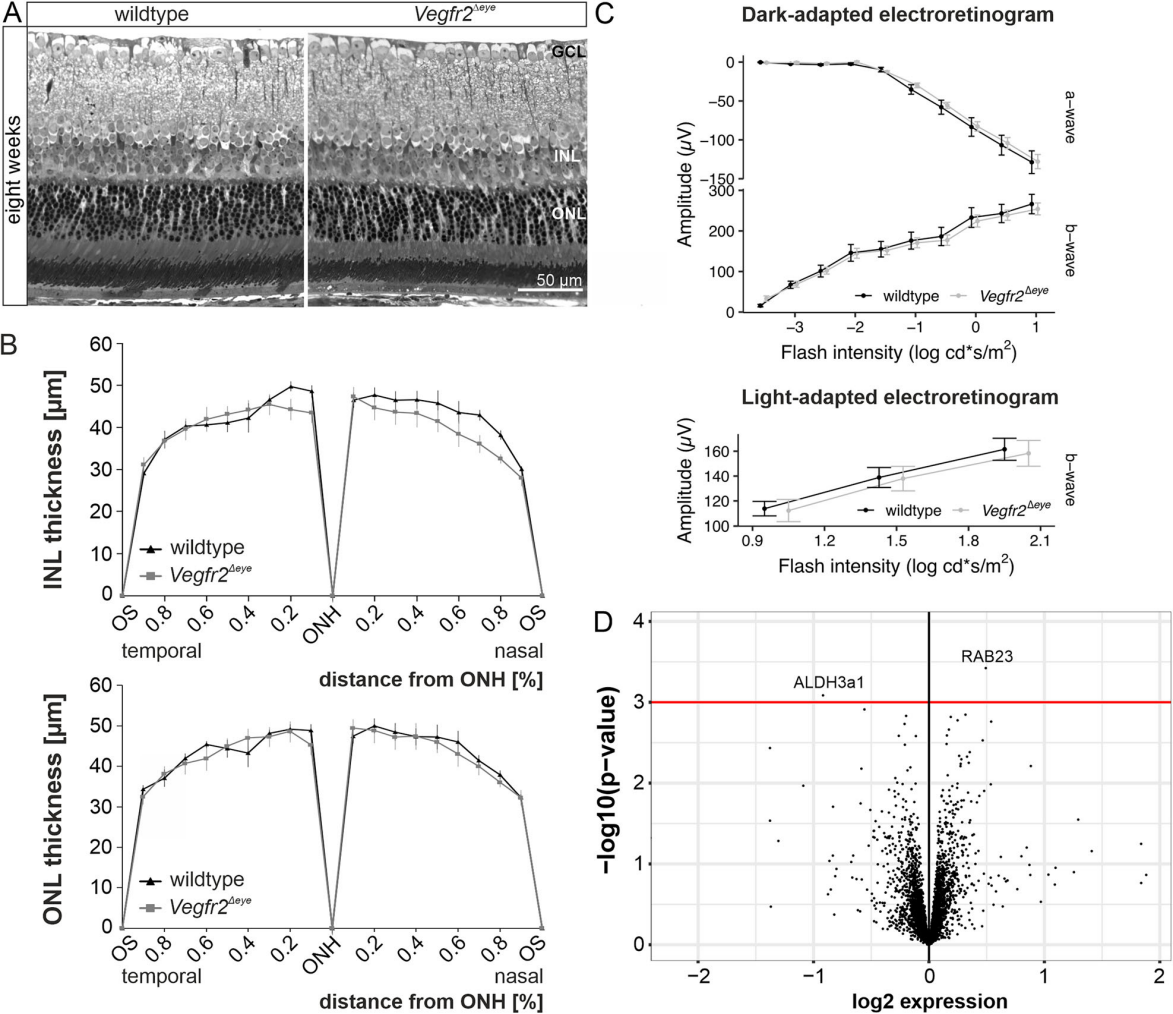
Retinal morphology, function and proteome of healthy *Vegfr2*^Δ*eye*^ and wildtype mice. **A** Richardson-stained, semithin sections of the central retina of six-weeks-old *Vegfr2*^Δ*eye*^ and wildtype animals. **B** Spider diagram illustrating the morphometric analyses of the INL and ONL thickness at defined measure points. Wildtype *n* = 7; *Vegfr2*^Δ*eye*^ = 6. Data are means ± SEM. Student’s t-test *p* > 0.05. **C** In vivo electroretinography (ERG) of *Vegfr2*^Δ*eye*^ and wildtype animals showing dark-adapted and light-adapted ERGs in eight-weeks-old mice with similar a- and b-wave amplitudes. Wildtype *n* = 4; *Vegfr2*^Δ*eye*^ = 5. **D** Volcano blot showing proteome analysis of six weeks old *Vegfr2*^Δ*eye*^ and wildtypes with 3729 identified retinal proteins of which two were significantly dysregulated, as indicated by their position above the red line (*p*-value < 0.001). RAB23 = Ras-related protein Rab-23; ALDH3A1 = aldehyde dehydrogenase 3 family member A1. GCL = ganglion cell layer; INL = inner nuclear layer; ONL = outer nuclear layer; OS = ora serrata; ONH = optic nerve head.

**Table 4:** Precise data of the individual experiments.

Figure	Analyses	Numbers
fig. 2A --- ---	*Vegfr2* expression (qPCR) *Vegfa120* (qPCR) *Vegfa164* (qPCR)	wildtype (DA): 1.00 ± 0.09, *n* = 6; wildtype (LD): 2.58 ± 0.24, *n* = 6; ***p = 0.0001 wildtype (DA): 1.00 ± 0.27, *n* = 8; wildtype (LD): 1.15 ± 0.22, *n* = 7; *p* = 0.67 wildtype (DA): 1.00 ± 0.26, *n* = 8; wildtype (LD): 1.40 ± 0.24, n = 7; *p* = 0.29
Fig. 2C	TUNEL-positive cells/mm^2^ ONL	wildtype: 526.00 ± 180.26, *n* = 15; *Vegfr2*^Δ*eye*^: 1653.25 ± 357.74, *n* = 11; **p = 0.005
Fig. 3A	*Lif* expression (qPCR)	wildtype (DA): 1.00 ± 0.27, *n* = 6; *Vegfr2*^Δ*eye*^ (DA): 2.43 ± 0.83, *n* = 6; wildtype (LD): 275.19 ± 96.87, *n* = 6; *Vegfr2*^Δ*eye*^ (LD): 418.46 ± 101.99, *n* = 6; *p* > 0.05
Fig. 3B	*Fgf2* expression (qPCR)	wildtype (DA): 1.00 ± 0.10, *n* = 6; *Vegfr2*^Δ*eye*^ (DA): 1.48 ± 0.42, *n* = 6; wildtype (LD): 4.18 ± 0.86, *n* = 6; *Vegfr2*^Δ*eye*^ (LD): 3.52 ± 0.85, *n* = 6; *p < 0.05
Fig. 3C	*Et2* expression (qPCR)	wildtype (DA): 1.00 ± 0.12, *n* = 6; *Vegfr2*^Δ*eye*^ (DA): 1.27 ± 0.23, *n* = 6; wildtype (LD): 11.61 ± 1.29, *n* = 6; *Vegfr2*^Δ*eye*^ (LD): 12.31 ± 2.11, *n* = 6; ***p < 0.001
Fig. 3D	*Gfap* expression (qPCR)	wildtype (DA): 1.00 ± 0.12, *n* = 5; *Vegfr2*^Δ*eye*^ (DA): 1.87 ± 0.54, *n* = 5; wildtype (LD): 10.51 ± 1.67, *n* = 6; *Vegfr2*^Δ*eye*^ (LD): 12.96 ± 1.58, *n* = 6, ***p < 0.001
Fig. 4C	AKT/pAKT protein expression	wildtype (DA): AKT: 1.00 ± 0.35, *n* = 8; wildtype (DA): pAKT: 0.48 ± 0.24, *n* = 8; wildtype (LD): AKT: 1.00 ± 0.14, *n* = 10; wildtype (LD): pAKT: 1.58 ± 0.22, *n* = 7; * *p* = 0.02
Fig. 4D	AKT/pAKT protein expression	*Vegfr2*^Δ*eye*^ (DA): AKT: 1.00 ± 0.14, *n* = 6; *Vegfr2*^Δ*eye*^ (DA): pAKT: 0.50 ± 0.19, *n* = 6; *Vegfr2*^Δ*eye*^ (LD): AKT: 1.00 ± 0.10, *n* = 11; *Vegfr2*^Δ*eye*^ (LD): pAKT: 1.04 ± 0.30, *n* = 7; *p* > 0.05, not significant
supplementary fig. 1A	*Vegfr2* expression (qPCR)	wildtype: 1.00 ± 0.10, *n* = 7; *Vegfr2*^Δ*eye*^: 0.42 ± 0.08, *n* = 7; *** *p* = 0.0008
supplementary Fig. 1B	VEGFR2 protein expression	retina + choroid: wildtype: 1.00 ± 0.11, *n* = 3 *Vegfr2*^Δ*eye*^: 0.38 ± 0.10, *n* = 3 **p = 0.002 neural retina: wildtype: 1.00 ± 0.16, *n* = 3 *Vegfr2*^Δ*eye*^: 0.41 ± 0.13, *n* = 3 **p = 0.007
supplementary fig. 2D	*Cd31* expression (qPCR)	wildtype: 1.00 ± 0.17, *n* = 11; *Vegfr2*^Δ*eye*^: 1.03 ± 0.13, *n* = 12; *p* > 0.05, not significant
supplementary fig. 2D	*Ng2* expression (qPCR)	wildtype: 1.00 ± 0.12, *n* = 11; *Vegfr2*^Δ*eye*^: 1.14 ± 0.09, *n* = 12; *p* > 0.05, not significant
supplementary fig. 2E	HIF1α protein expression	wildtype: 1.00 ± 0.12, *n* = 6; *Vegfr2*^Δ*eye*^: 0.89 ± 0.32, *n* = 7; *p* > 0.05, not significant

Next, we studied the retinal morphology of *Vegfr2*^Δ*eye*^ and wild-type littermates at the ages of six weeks and six months. Here, we did not observe obvious morphological alterations (Fig. 1A (six weeks old) and supplementary fig. 1D (six months old)). Morphometric analyses of the thickness of the inner and outer nuclear layer (INL, ONL) on semithin sections of six-week-old and six-month-old animals did not show significant differences between *Vegfr2*^Δ*eye*^ and wildtype littermates, despite one slightly thicker INL measure point in six-month-old *Vegfr2*^Δ^^eye^ retinae (Fig. 1B (six weeks old), supplementary fig. 1E (six months old)). Accordingly, functional electroretinogram (ERG) analysis did not reveal alterations when comparing rod- as well as cone-dominated waveforms of eight-week-old *Vegfr2*^Δeye^ and wildtype retinae (Fig. 1C and supplementary fig. 1F). In addition, three-month-old *Vegfr2*^Δeye^ and wildtype animals demonstrated a regular fundus (supplementary fig. 2A).

Intriguingly, although VEGFR2 is a key component of (retinal) angiogenesis [45], its deletion after completed retinal vascular development showed no abnormalities of the retinal vasculature, as observed by in vivo imaging (supplementary fig. 2A). Accordingly, ocular sections from six-week-old animals perfused with FITC-dextran (supplementary fig. 2B) and FITC-dextran perfused retinal flatmounts at the age of three months (supplementary fig. 2C) showed a regular organization of the three retinal vascular plexus and the choriocapillaris. Moreover, retinal expression of the endothelial cell marker cluster of differentiation 31 (*Cd31*) and the pericyte marker neural/glial antigen 2 (*Ng2*) did not show significant differences when comparing their retinal expression in *Vegfr2*^Δ*eye*^ and wildtypes (supplementary fig. 2D; Table 4). We furthermore analyzed the retinal expression levels of hypoxia-inducible factor 1-alpha (HIF1α), which is a very sensitive marker of tissue hypoxia as it is degraded by the proteasome under normoxic conditions, but stabilized upon hypoxia [46]. We did not observe significant differences in its expression when comparing six-week-old *Vegfr2*^Δ^^eye^ and wild-type (supplementary fig. 2E; Table 4) retinae, suggesting comparable vascularization and oxygenation between the two groups. In addition, immunohistochemical staining for plasmalemmal vesicle-associated protein (PV1), which is a marker for fenestrated endothelium [47], showed a regular and continuous staining pattern of the choriocapillaris at the age of six weeks (supplementary fig. 2F).

### *Vegfr2* deletion and the retinal proteome

Next, we used spectrometric analyses (LC-MSMS-based proteomics) to investigate the impact of post-developmental *Vegfr2* deletion on the retinal proteome in *Vegfr2*^Δ*eye*^ and wild-type animals. We identified a total of 3729 proteins in the retinae, none of which were dysregulated between *Vegfr2*^Δeye^ and wildtype after multiple hypothesis correction. When slightly relaxing the cut-off criteria to *p*=0.001, we identified two dysregulated proteins, of which one was down-regulated, and one was up-regulated (Fig. 1D). Specifically, Ras-related protein Rab-23 (RAB23) was approximately. 40% upregulated, and aldehyde dehydrogenase 3 family member A1 (ALDH3A1) was approximately. 48% downregulated in *Vegfr2*^Δ*eye*^ retinae (Fig. 1D). Taken together, the deletion of VEGFR2-mediated signaling in the adult eye had an almost negligible effect on the retinal proteome, consistent with the morphological and functional readouts described above.

### *Vegfr2* deficiency causes significant structural alterations in the light-exposed retinae

**Fig. 2:**
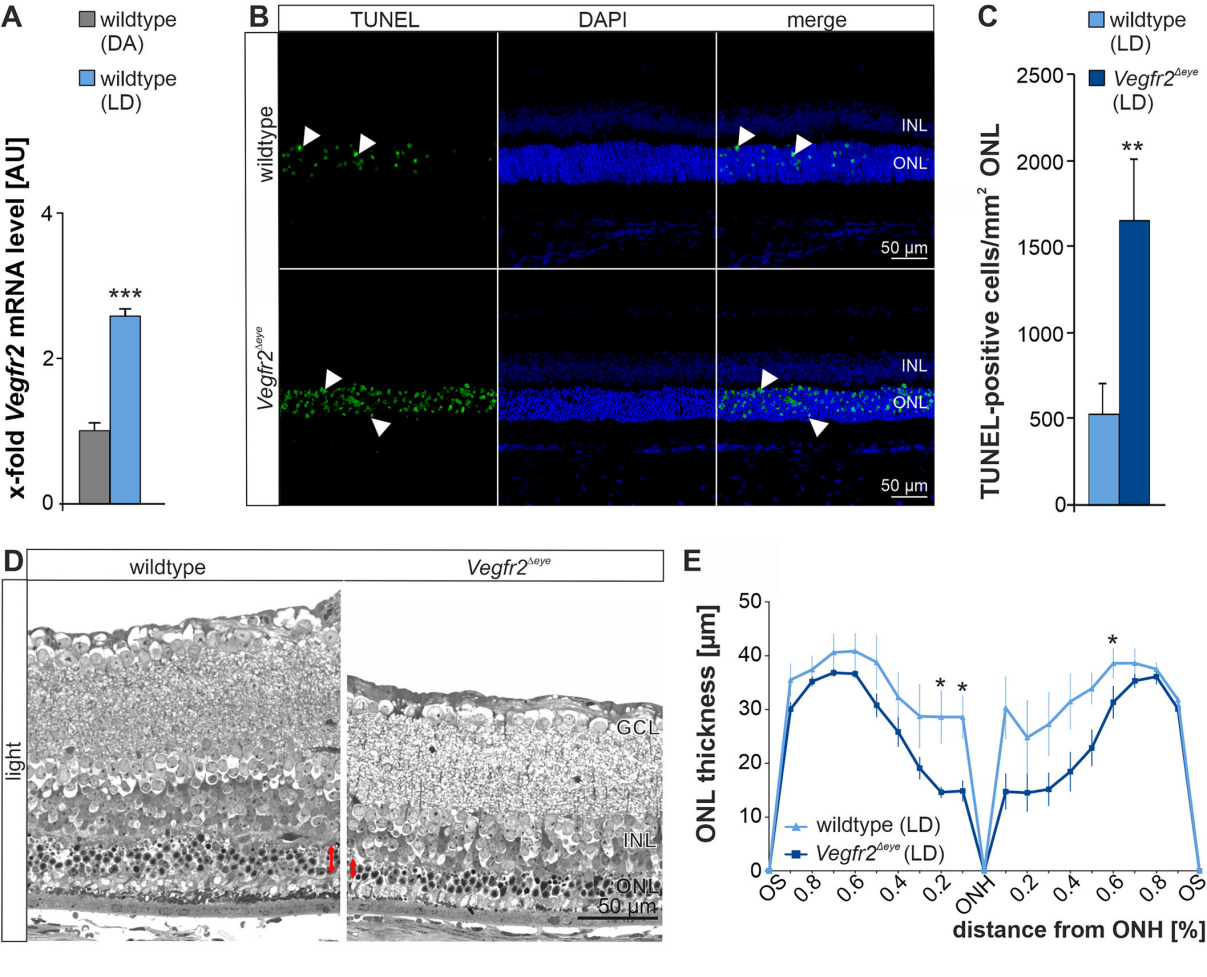
Light-induced neurodegeneration in *Vegfr2*^Δ*eye*^ and wild-type mice. **A** qPCR analyses for mRNA of retinal *Vegfr2* in six-week-old wildtype animals 6 h following light-exposure (*n* = 6). **B** Mid-horizontal sections of six-week-old, light-exposed, TdT-mediated dUTP-biotin nick end (TUNEL)-labeled central retinae. Following light exposure, numerous TUNEL-positive cells (green, arrowheads) cells were detected in the ONL. Cell nuclei were stained with DAPI (blue). **C** Quantification of TUNEL-positive cells/mm^2^ ONL. (Wildtype *n* = 15; *Vegfr2*^Δ*eye*^ *n* = 11). **p = 0.005. **D** Richardson-stained, semithin sections of the central retina of eight-weeks-old, light-exposed *Vegfr2*^Δ*eye*^ and wildtype retinae showing a thinner ONL (red arrows) and shortened photoreceptor outer and inner segments in both eyes, which was however considerably thinner in the *Vegfr2*^Δ*eye*^ animal compared the wildtype. **E** Spider diagram illustrating the ONL thickness following light exposure at defined measurement points. Wildtype *n* = 6; *Vegfr2*^Δ*eye*^ = 7. Data are means ± SEM. Student’s t-test. *p ≤ 0.05, **p ≤ 0.01. *Vegfr2* = vascular endothelial growth factor receptor 2; GCL = ganglion cell layer; INL = inner nuclear layer; ONL = outer nuclear layer; OS = ora serrata; ONH = optic nerve head.

In the next step, we aimed to investigate whether VEGFR2-mediated signaling might have an impact on the survival of retinal neurons under stressed conditions. To this end, we induced photoreceptor degeneration by using the light damage model, resulting in similar phenotypes to those observed in human patients affected by the dry, geographic form of AMD [20]. Intriguingly, we detected a significant increase of *Vegfr2* expression following exposure to cool white light in wildtype retinae (Fig. 2A; Table 4). *Vegfa120* expression remained unchanged, whereas *Vegfa164* expression showed a slight, non-significant increase (*p* = 0.29; Table 4). Furthermore, following light damage, we observed a significant increase of apoptotic, TUNEL-positive cells in the ONL (Fig. 2B, C) in *Vegfr2*^Δ*eye*^ retinae (**p = 0.005, Table 4) compared to light-exposed wildtype retinae. To investigate whether the observed increase in apoptosis would result in morphological differences, we analyzed the thickness of the ONL 14 days after light exposure on semithin sections. The central part of wildtype retinae demonstrated five to six rows of remaining nuclei in the ONL (Fig. 2D, red arrow). In contrast, the thickness of the ONL in light-exposed *Vegfr2*^Δ*eye*^ retinae was remarkably reduced, with only approximately three layers of nuclei left (Fig. 2D, red arrow). This resulted in a significant thinning of the ONL, as shown in the spider diagrams of light-exposed *Vegfr2*^Δ*eye*^ (*p ≤ 0.05) compared to light-exposed wildtype retinae (Fig. 2E) and was functionally accompanied by slightly reduced amplitudes of the rod- and cone- driven electroretinogram in light-exposed *Vegfr2*^Δ*eye*^ retinae (supplementary fig. 3).

### *Vegfr2* deficiency does not alter the expression of neuroprotective factors

**Fig. 3:**
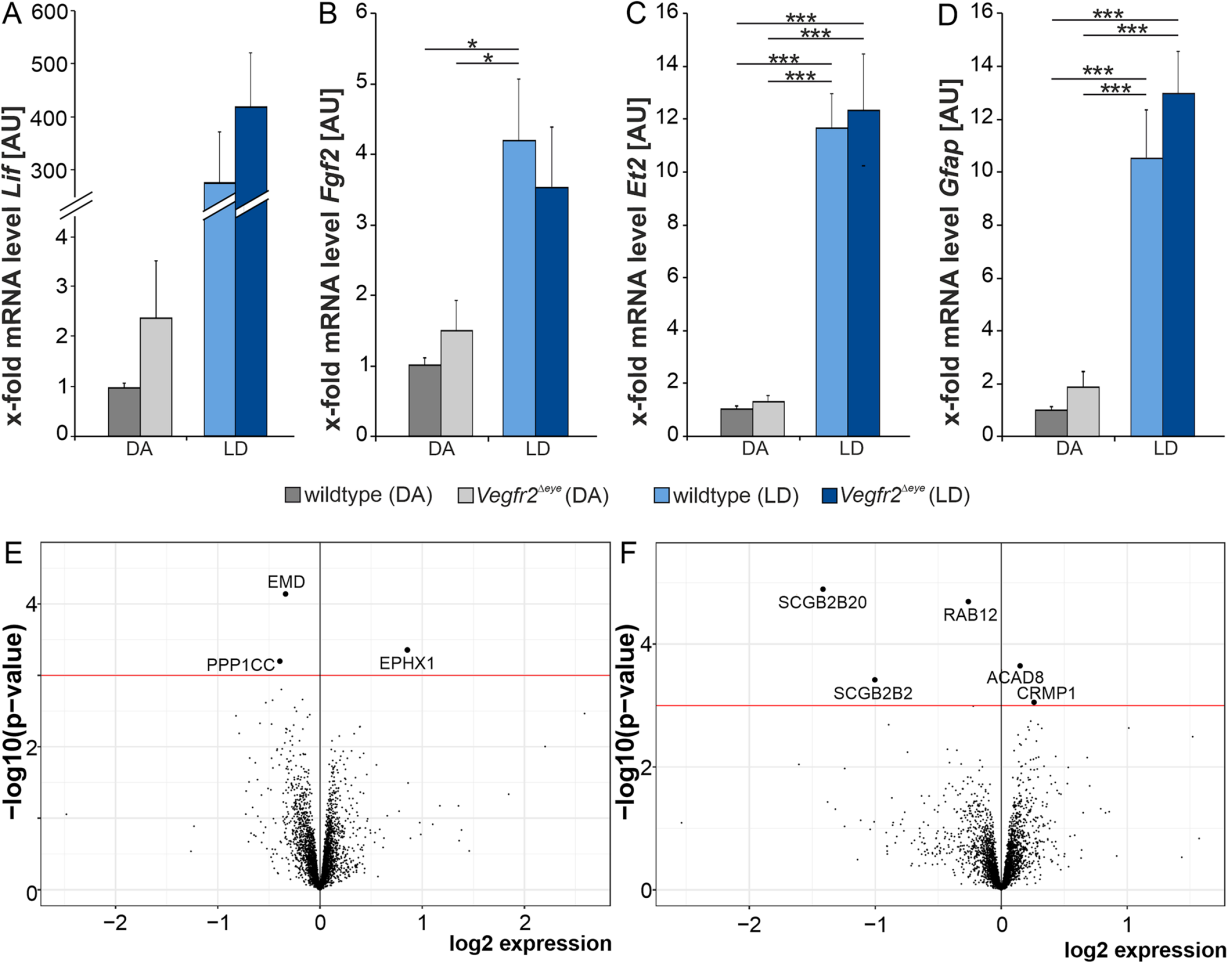
Early response to light damage in *Vegfr2*^Δ*eye*^ and wildtype retinae. qPCR analyses for *Lif* (**A**)*, Fgf2* (**B**)*, Et-2* (**C**) and *Gfap* (**D**) mRNA in six-week-old wildtype and *Vegfr2*^Δ*eye*^ retinae without (dark-adapted, DA) and 6 h following light damage (LD). Data are means ± SEM, one-way ANOVA (Bonferroni post hoc test). *n* ≥ 5 (for details see Table 4); *p ≤ 0.05, ***p ≤ 0.001. Volcano blot showing proteome analysis of six weeks old dark-adapted compared to light-exposed wildtypes (**E**) or *Vegfr2*^Δ*eye*^ (**F**) retinae. Significantly dysregulated proteins are indicated by their position above the red line (*p*-value < 0.001). Wildtype *n* = 6; *Vegfr2*^Δ*eye*^ = 6. *Lif* = leukemia inhibitory factor*, Fgf2* = fibroblast growth factor 2; *Et2* = endothelin 2; *Gfap* = glial fibrillary acidic protein; EMD = emerin; PPP1CC = protein phosphatase 1 catalytic subunit gamma; upregulated: EPHX1 = epoxide hydrolase 1; SCGB2B20 = secretoglobin family 2B member 20, SCBG2B2 = secretoglobin family 2B member 2, RAB12 = ras-related protein Rab-12; upregulated: ACAD8 = acyl-coA dehydrogenase family member 8, CRMP1 = collapsin response mediator protein 1.

We are now focused on molecular mechanisms that might contribute to the enhanced neurodegeneration in *Vegfr2*^Δ*eye*^ animals. First, we analyzed the retinal expression levels of leukemia inhibitory factor (*Lif)*, fibroblast growth factor 2 *(Fgf2)* and endothelin 2 *(Et2)*, before and 6 h after light exposure (Fig. 3A–C; Table 4), as these factors act neuroprotectively and have been described to be upregulated following retinal degeneration, including light-induced photoreceptor degeneration [26, 48–51]. *Lif* was barely detectable in the retinae of dark-adapted *Vegfr2*^Δ*eye*^ and wildtype littermates. Yet, light-exposed *Vegfr2*^Δ*eye*^ and wildtype retinae showed a robust increase of retinal *Lif* expression (*p* > 0.05) (Fig. 3A; Table 4). When analyzing the retinal expression levels of *Fgf2* (Fig. 3B; Table 4) and *Et2* (Fig. 3C; Table 4), we did not observe significant alterations in their expression levels when comparing dark-adapted *Vegfr2*^Δ*eye*^ and wildtype retinae. Following light exposure, both genotypes showed a remarkable increase in their retinal *Fgf2* and *Et2* expression compared to their dark-adapted littermates (*Fgf2*: wildtype: *p* = 0.01, *Vegfr2*^Δ*eye*^: *p* > 0.05; *Et2*: wildtype: *p* < 0.001, *Vegfr2*^Δ*eye*^: *p* < 0.001), which was however not significantly altered within the light-exposed groups (Fig. 3B, C; Table 4). The intermediate filament glial fibrillary acidic protein (*Gfap*) is expressed in astrocytes and upregulated in astrocytes and Müller cells upon neurodegenerative insults [52–54]. We found that *Gfap* expression levels were not significantly altered between *Vegfr2*^Δ*eye*^ and wildtype retinae (Fig. 3D; Table 4). However, after light exposure, *Gfap* expression was significantly increased compared to the dark-adapted groups (wildtype: *p* = 0.0004, *Vegfr2*^Δ*eye*^: *p* = 0.0006) (Fig. 3D; Table 4), yet we detected no statistically significant difference between the two light-exposed groups.

### *Vegfr2* deficiency and the retinal proteome under light-induced photoreceptor degeneration

**Fig. 4:**
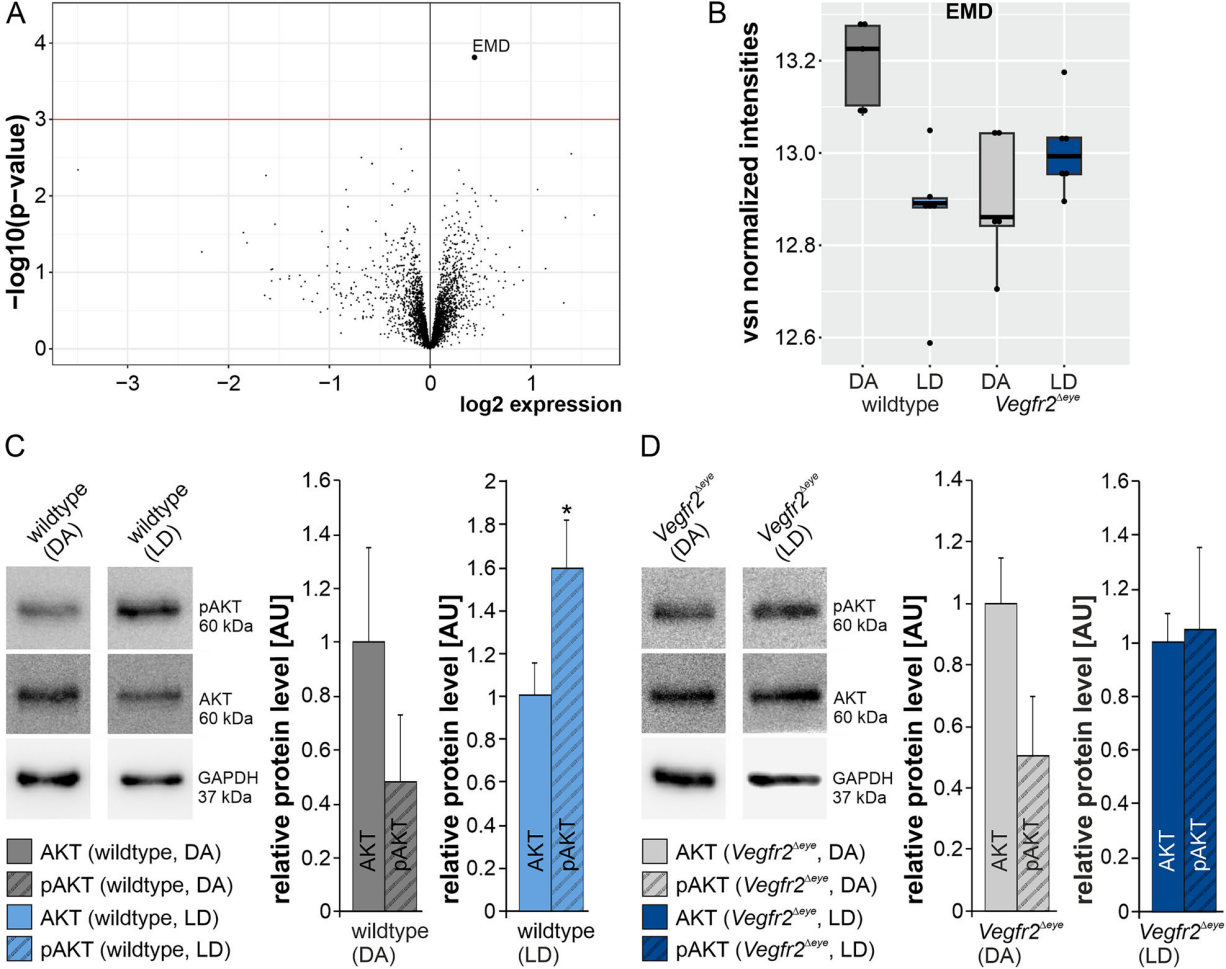
The proteome and AKT signaling in *Vegfr2*^Δ*eye*^ and wildtype retinae - with and without light exposure. **A** Volcano blot of six weeks old mice showing the dysregulation analysis of the interaction of the two main factors: genotype (*Vegfr2*^Δ*eye*^ or wildtype) and treatment (LD vs. DA). Only EMD (emerin) was significantly dysregulated, as indicated by its position above the red line (*p*-value < 0.001). n = 6 for both LD groups and n = 5 for both DA groups. **B** Normalized EMD intensities. Western blot analyses and corresponding densitometric analyses for retinal AKT and phosphorylated (p) AKT in six weeks old wildtype (**C**) and *Vegfr2*^Δ*eye*^ (**D**) dark-adapted (DA, wildtype: *n* = 8; *Vegfr2*^Δ*eye*^ *n* = 6) and 6 h following light exposure (LD, wildtype: AKT: *n* = 10, pAKT: *n* = 7; *Vegfr2*^Δ*eye*^: AKT: *n* = 11, pAKT: *n* = 7). The reference protein glyceraldehyde 3-phosphate dehydrogenase (GAPDH) was used as loading control. Data are means ± SEM. Student’s t-test. *p ≤ 0.05. AKT = proteinkinase B; pAKT = phosphorylated AKT; DA = dark-adapted; LD = light damage.

To further study the immediate response to light damage, we analyzed the retinal proteome 6 h after damage. Here, we detected three dysregulated proteins (downregulated: EMD = emerin, PPP1CC = protein phosphatase 1 catalytic subunit gamma; upregulated: EPHX1 = epoxide hydrolase 1) when comparing light-exposed wildtype to dark-adapted wildtype retinae (Fig. 3E). We also analyzed protein expression of light-exposed *Vegfr2*^Δ*eye*^ and dark-adapted *Vegfr2*^Δ*eye*^ retinae and found five dysregulated proteins (downregulated: SCGB2B20 = secretoglobin family 2B member 20, SCBG2B2 = secretoglobin family 2B member 2, RAB12 = ras-related protein Rab-12; upregulated: ACAD8 = acyl-CoA dehydrogenase family member 8, CRMP1 = collapsin response mediator protein 1) (Fig. 3F). When we analyzed the statistical interaction of VEGF signaling and light damage, in other words the modulation of the light damage response due to the presence or absence of VEGF signaling, we found only emerin (EMD) to be significantly dysregulated (Fig. 4A). EMD expression levels across the four groups revealed a downregulation of EMD following light damage in wildtype retinae, whereas this effect was absent in light-exposed *Vegfr2*^Δ*eye*^ retinae, which instead showed a trend towards upregulation (Fig. 4B).

### *Vegfr2* deficiency inhibits AKT activation under light-induced photoreceptor degeneration

Emerin is likely involved in the phosphorylation of protein kinase B (AKT) [55], a signaling pathway that regulates a wide range of cellular processes, including regulation of cell proliferation, survival, and metabolism [56]. Furthermore, AKT signaling is described as a potent neuroprotective mediator [26, 57, 58]. To study this, we analyzed AKT and its active, phosphorylated form (pAKT), in the retinae of *Vegfr2*^Δ*eye*^ and wildtypes. AKT and pAKT signals were detectable as distinct bands that migrated at their expected molecular weight of 60 kDa in retinal proteins of light-exposed and dark-adapted animals (Fig. 4C, D). Densitometric analyses showed no significant differences between AKT and pAKT expression in wildtype (Fig. 4C; Table 4) and *Vegfr2*^Δ*eye*^ (Fig. 4D; Table 4) retinae without light exposure. However, following light exposure, we observed a significant increase in the expression of pAKT compared to AKT in wildtype retinae (*p* = 0.02, Fig. 4C), an effect that was essentially absent in light-exposed *Vegfr2*^Δ*eye*^ retinae (Fig. 4D).

### Vegfr2 expression in the individual retinal cell populations in humans

**Fig. 5:**
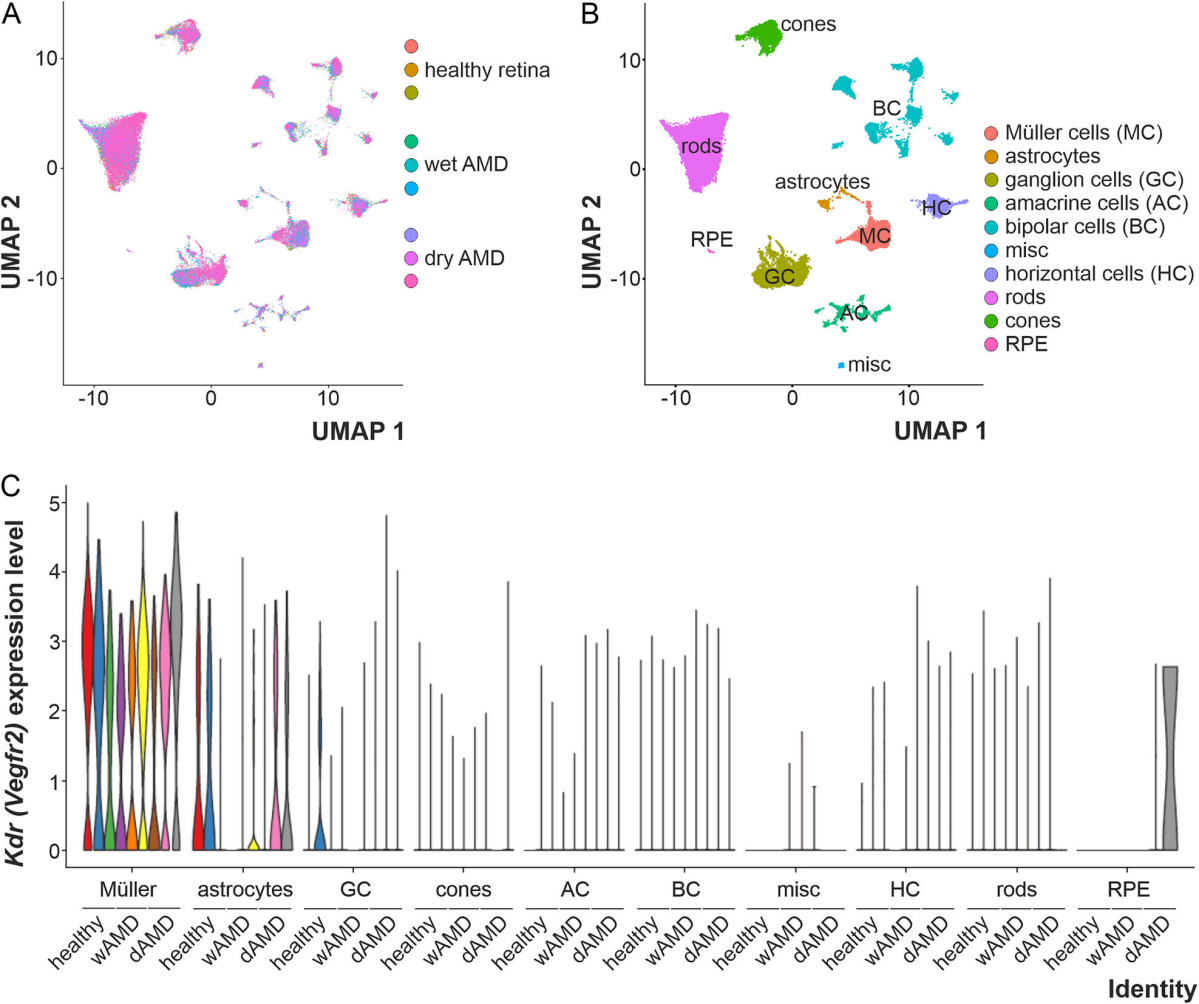
Single-nuclei sequencing data of human healthy retinae, wet and dry AMD. Single nuclei RNA expression data from healthy human donor retinae, wet and dry AMD retinae were used to cluster cells (**A**) and assign cell types (**B**) as described in the methods section. Uniform manifold approximation and projection (umap) dimensions 1 and 2 for each cell type are shown. **C** Violin blots reflecting the expression levels of *Kdr (Vegfr2)* in the identified retinal cell populations in healthy, wet AMD and dry AMD conditions are shown. Healthy human donors *n* = 3, wAMD *n* = 3, dAMD *n* = 3. Müller = Müller cells, GC= ganglion cells, AC= amacrine cells, BC= bipolar cells, HC= horizontal cells, RPE= retinal pigment epithelium. *Vegfr2* = vascular endothelial receptor 2, *Kdr* = Kinase Insert Domain Receptor, wAMD = wet AMD, dAMD = dry AMD.

As briefly mentioned before, there are clinical observations and reports that anti-VEGF therapy in patients with neovascular and geographic (dry) AMD in the same eye might increase the development of geographic atrophy [17]. Clinical anti-VEGF therapy ultimately amounts to an inhibition of the VEGF signaling pathway that primarily acts through VEGFR2 activation. Accordingly, we investigated the specific retinal cell types expressing VEGFR2 in the human retina to identify which cell population(s) might contribute to the observed effects. Additionally, we examined the potential alterations in cell-type-specific VEGFR2 expression in the context of neovascular and/or geographic (dry) AMD. To this end, we analyzed a publicly available single-nuclei sequencing (snSeq) data set (GSE221042, [44]) of retinal samples from healthy human donors and individuals with neovascular AMD and intermediate dry AMD. Using the state-of-the-art bioinformatic alevin-Fry and Seurat frameworks, we identified 10 cell types. The expression of well-established marker genes [59–61] for each cluster confirmed the accurate assignment of cell types and showed excellent separation between them (Fig. 5A, B and supplementary fig. 4). In summary, rods, cones, astrocytes, Müller cells, retinal ganglion (RG) cells, amacrine cells (AC), bipolar cells (BC), horizontal cells (HC) and retinal pigmented epithelial (RPE) cells were identified.

When analyzing the expression of *Vegfr2 (Kdr)*, *Vegfr1 (Flt1)*, and *Vegfa* in the individual retinal cell populations, we found *Vegfr1* and *Vegfr2* predominantly expressed in macroglial cells such as astrocytes and Müller cells (Fig. 5C and supplementary fig. 5A). Accordingly, immunohistochemical staining for VEGFR2 and the Müller cell marker glutamine synthetase showed clear co-localization in our samples (supplementary fig. 1C). *Vegfa* was expressed in all identified retinal cell types (supplementary fig. 5B). We did not detect a prominent expression of *Vegfr2* in photoreceptors (rods and cones), indicating that the (neuro-)degenerative effect upon *Vegfr2* deletion, which we observed in this study, or which is reported to occur due to the clinical anti-VEGFA treatments in humans, is mediated through Müller cells and/or astrocytes.

## Discussion

Based on our results, we conclude that deficiency of retinal VEGF signaling affects the retinal proteome only very mildly and does not result in obvious morphological alterations and impaired function in the post-developmental, healthy retina. However, under pathological conditions such as in light-induced photoreceptor degeneration, a well-established damage model mimicking certain aspects of the geographic form of AMD, the deletion of VEGF signaling significantly promotes photoreceptor degeneration. Intriguingly, on a molecular level, this does not involve altered expression of neuroprotective factors such as *Lif, Et2*, or *Fgf2*, but increased expression of emerin and lack of phosphorylation of AKT in retinae with VEGF deficiency. Finally, single-nuclei sequencing data of human retinal samples of healthy individuals, dry- and wet AMD patients, suggest a VEGF-dependent crosstalk from glial cells to photoreceptors that acts neuroprotectively.

### Deletion of *Vegfr2* in the adult, healthy retina

Since a systemic deletion of components of the VEGF signaling pathway results in embryonic lethality [62], we used a tamoxifen-inducible Cre/loxP-based approach to delete *Vegfr2* in the adult, murine eye. When comparing the relative *Vegfr2* mRNA expression levels, we observed a constant, comparable and significant reduction of *Vegfr2* between the individual retinae of the treated animals. In fact, there are conflicting data regarding the role of VEGF signaling in the adult retina. Systemic injection of an adenoviral vector expressing soluble VEGFR1 (sFlt1) caused a significantly increased apoptosis in the INL and ONL concomitant with a reduced thickness of the INL and ONL and retinal function [21]. The conditional deletion of VEGFA in adult mouse RPE cells resulted in ablation of the choriocapillaris, dysfunction of cone photoreceptors, but relatively comparable retinal thicknesses as determined by optical coherence tomographic (OCT) analysis up to seven months [63]. Brown Norway rats that received three intraocular injections of an antibody against human VEGFR2, which is known to cross-react with rat VEGFR2, did not show functional deficits or a thinning of OCT scans/the ONL four weeks after VEGFR2 inhibition [64]. However, mice that did express only VEGFA isoform 188 constitutively, which is known to be sequestered on the cell surface or in the extracellular matrix (in contrast to the diffusible VEGFA isoforms 120 and 164), exhibited a progressive degeneration of the choriocapillaris, RPE abnormalities, photoreceptor apoptosis and impaired retinal function - but only in older mice [65]. To our understanding, the different extent of neurodegeneration after VEGF signaling inhibition in the adult eye depends most likely on the degree of VEGF signaling inhibition. Based on the data of our study, we conclude that the reduction in retinal VEGFR2-mediated signaling up to 58% in the adult and otherwise healthy retina has no negative effects on its structure and function.

Accordingly, our approach to analyze the impact of a VEGFR2 deletion on the retinal proteome revealed only two dysregulated proteins in retinae with a deletion of *Vegfr2*. Ras-related protein Rab-23 (RAB23) is a member of the Rab GTPase family whose functions include, amongst others, the formation of vesicles, intracellular transport processes, including autophagy, and signal transduction [66]. Intriguingly, increased autophagic processes were also observed under light-exposure conditions [67]. RAB23 is furthermore known for its regulatory function of the Hedgehog signaling pathway, which also plays a role in retinogenesis and the maintenance of tissue structures [68–70]. Aldehyde dehydrogenase 3 family member A1 (ALDH3A1) belongs to the family of aldehyde dehydrogenases, which are enzymes that are involved in the cellular maintenance and homeostasis by processing both endogenous and exogenous reactive compounds [71]. ALDH3A1 preferentially oxidizes aromatic and medium-chain (6 carbons or more) saturated and unsaturated aldehyde substrates and thus protects inner ocular tissues from ultraviolet radiation and reactive oxygen-induced damage [72]. Retinal single-cell data show that ALDH3A1 is expressed in Müller cells, endothelial cells and very sparsely in rod photoreceptors and bipolar cells (The human protein atlas, https://v22.proteinatlas.org/, accessed Feb. 2025). Intriguingly, and in line with our findings, ALDH3A1 was also among the most significantly downregulated proteins in Müller glia following deletion of the membrane-bound interleukin-6 receptor (IL-6Rα) [73], a condition in which the expected IL-6-induced VEGF production did not occur [73]. Thus based on the data of this study and our data, we state that ALDH3A1 expression is downstream of and regulated through VEGF signaling. Reduced levels of ALDH3A1 have also been associated with increased oxidative damage and reduced cell proliferation [71, 74]. Therefore, it is also reasonable to assume that the decreased ALDH3A1 expression levels observed in our model, enhanced vulnerability of the retinal cells towards oxidative species in particular since light-exposure induces reactive oxygen species, generated by bleaching of rhodopsin or from compounds in the RPE [19].

### Deletion of *Vegfr2* in pathologic conditions

In the second part of the study, we investigated the role of VEGFR2-mediated signaling in photoreceptor survival under stress conditions. The rationale behind this was to mimic the situation of patients treated with anti-VEGF therapy in the clinic, such as patients affected by neovascular AMD. Of note, the tamoxifen-inducible system that we used allowed the deletion of *Vegfr2* in the adult retina, thus a situation quite comparable to patients in the clinic receiving anti-VEGF therapy. To induce cellular stress, we used the light-damage model, a well-established damage model that results in photoreceptor degeneration [26, 29]. The light-damage model is furthermore considered as a model mimicking certain aspects of the geographic form of AMD [20]. We observed a significantly higher number of apoptotic photoreceptor cells concomitant with a significantly thinner ONL following light exposure in retinae with a deletion of *Vegfr2*. Accordingly, published data show that VEGFA treatment of primary retinal ganglion cells in vitro and in vivo promoted their survival in two different damage models (in vitro: H_2_O_2_ (10 μmol/L); staurosporine (1 μmol/L), in vivo: staurosporine and magnetic bead ocular hypertension model), an effect that was furthermore shown to act via the PI3K/AKT signaling axis [75]. Our data confirm those findings for photoreceptor cells under stress conditions using the light damage model. In this context, it is particularly noteworthy that there is clinical evidence suggesting an association between anti-VEGF therapy and the progression of geographic atrophy in human AMD patients [17] which our data clearly support. However, a study reports that three injections of recombinant VEGFA165 in the vitreous of mice with a hereditary, progressive degeneration of photoreceptors (*Rd10* [76]) did not improve their retinal function [64]. In our opinion, this may be attributable to the more aggressive nature of retinal degeneration in the *Rd10* model compared to the light-induced damage that we used in our study. Consequently, three intravitreal VEGFA165 injections might not be sufficient to exert a lasting neuroprotective effect capable of counteracting the profound degenerative impact of the *Rd10* mutation.

Our approach to investigating the immediate and early changes in the expression of retinal proteins by mass spectrometry identified a few dysregulated proteins after light exposure. Yet, we still detected some dysregulated proteins, of which emerin caught our attention in particular. Emerin is an ubiquitously expressed protein of the inner nuclear membrane, a cellular compartment that is considered a subdomain of the endoplasmic reticulum [77, 78]. The reduced expression of emerin in light-damaged wildtype retinae compared to dark-adapted wildtype retinae and the absence of this effect in *Vegfr2*^Δ*eye*^ retinae is of particular interest for our project since, in line with our data, a study showed that knockdown of Emerin induced phosphorylation of ERK and AKT and inhibited hydrogen peroxide-induced apoptosis in HeLa cells [55].

Accordingly, our western blot analyses for AKT and phosphorylated AKT (pAKT) in retinal proteins from light-exposed animals showed a significantly higher level of pAKT in retinae of light-exposed wildtype animals, an effect that was essentially absent in *Vegfr2-*deficient animals. In addition to our observation in light-exposed wildtype retinae, others have also described the activation of AKT in response to light damage [79]. Moreover, the work of Fu and colleagues showed that inhibition of *Vegfr2* in Müller cells leads to significantly lower activation of AKT under high glucose stress compared to similarly treated wildtypes [80]. AKT is well described to act in a neuronal survival cascade [26, 81]. Thus, the missing activation of AKT in *Vegfr2*-deficient retinae suggests that under cell-stress conditions such as light exposure, the deletion of the VEGF signaling pathway reduces the activity of the PI3K/pAKT pathway. Hence, we propose that the exacerbated neurodegeneration observed in *Vegfr2*-deficient animals is attributable to the absence of activation of the neuroprotective AKT signaling pathway.

### *Vegfr2* expression in the human retina in health and disease

Reports indicate that anti-VEGFA therapy in patients with geographic, dry AMD promotes the development of atrophic zones [17]. Considering that VEGFR2 is the main receptor for VEGF-mediated signaling [9], anti-VEGFA therapy in humans primarily inhibits VEGF-mediated signaling in cells harboring *Vegfr2*. Our single nuclei analysis shows that astrocytes and Müller cells predominantly express *Vegfr2*. From a mechanistic perspective, clinically administered anti-VEGFA therapy closely resembles the functional ablation of VEGF signaling that we achieved by deleting *Vegfr2*. Accordingly, the observed increased degeneration of photoreceptors following light exposure strongly implies a VEGF-driven, paracrine neuroprotective mechanism proceeding from astrocytes/Müller cells. In view of that, recently published data demonstrated a significant upregulation of GFAP in retinae of mice subjected to oxygen-induced retinopathy, while inhibition of VEGF signaling markedly reduced GFAP expression [82], suggesting that VEGF signaling also modulates the reactivity of glial cells. Accordingly, in vitro co-cultures of anti-VEGFA-pretreated Müller cells with 661 W photoreceptor cells affected neurodegeneration of 661 W cells under hypoxic conditions [82]. Moreover, mice with an inhibition of *Vegfr2* specifically in Müller cells exhibited impaired retinal function and degeneration of retinal neurons, including photoreceptors, in a mouse model of diabetes [80]. In summary, these data, together with the data we present in this study, strongly support the concept of a VEGF signaling-dependent neuroprotective crosstalk from Müller cells to photoreceptors. Thus, the stimulation of VEGF signaling in Müller cells might be a promising approach to attenuate the degeneration of photoreceptors in diseases such as age-related macular degeneration, diabetic retinopathy or retinitis pigmentosa. Moreover, in view of the resulting increased degeneration in the event of cellular stress (light damage in mice, dry AMD in humans), we propose a careful consideration of the cost-benefit ratio of anti-VEGFA therapy in the clinical context. This is particularly relevant for patients with dry AMD and comorbidities like choroidal neovascularization in the same eye. We believe that targeting solely endothelial cells using a cell-specific anti-VEGFA therapy would prevent the risk of undesirable side effects, such as an increase in retinal degeneration.

## Supplementary information

Summary of Supplemental Material

Supplementary figure 1: Deletion of ocular Vegfr2 in healthy animals: retinal morphology and function

Supplementary figure 2: Retinal vasculature of Vegfr2^Δeye^ and wild-type animals

Supplementary figure 3: ERG of light-damaged animals

Supplementary figure 4: Identification of cell types in the human retina following snRNA sequencing

Supplementary figure 5: Expression of Vegfa and Vegfr1 in single nuclei sequencing data of human healthy retinae, wet AMD and dry AMD.

Unedited western blot gels

## Data Availability

The mass spectrometry proteomics data have been deposited to the ProteomeXchange Consortium via the PRIDE [35] partner repository with the dataset identifier PXD066263.
