# Supplementary material for: VEGFR2 deletion increases susceptibility to photoreceptor degeneration through glial-neuronal interaction
Source: Cell Death Dis. 2026 Jun 11;17(1):564. doi: 10.1038/s41419-026-08963-z (PMC13260824; doi:10.1038/s41419-026-08963-z)
Supplement: Supplementary file 1 — Summary of Supplemental Material [file 41419_2026_8963_MOESM1_ESM.docx]

# VEGFR2 deletion increases susceptibility to photoreceptor degeneration through glial-neuronal interaction

Christina B. Bielmeier*^2,4^, Sabrina I. Schmitt*^2^, Verena Lehr^4^, Anita Grundl^2^, Andrea E. Dillinger^1^, Herbert Jägle^3^, Christine von Toerne^5^, Stefanie M. Hauck^5^, Süleyman Ergün^1,4,7^, Ernst R. Tamm^2^, Anja Schlecht^1,4^, Andreas Neueder^6^ and Barbara M. Braunger^1,2,4#^


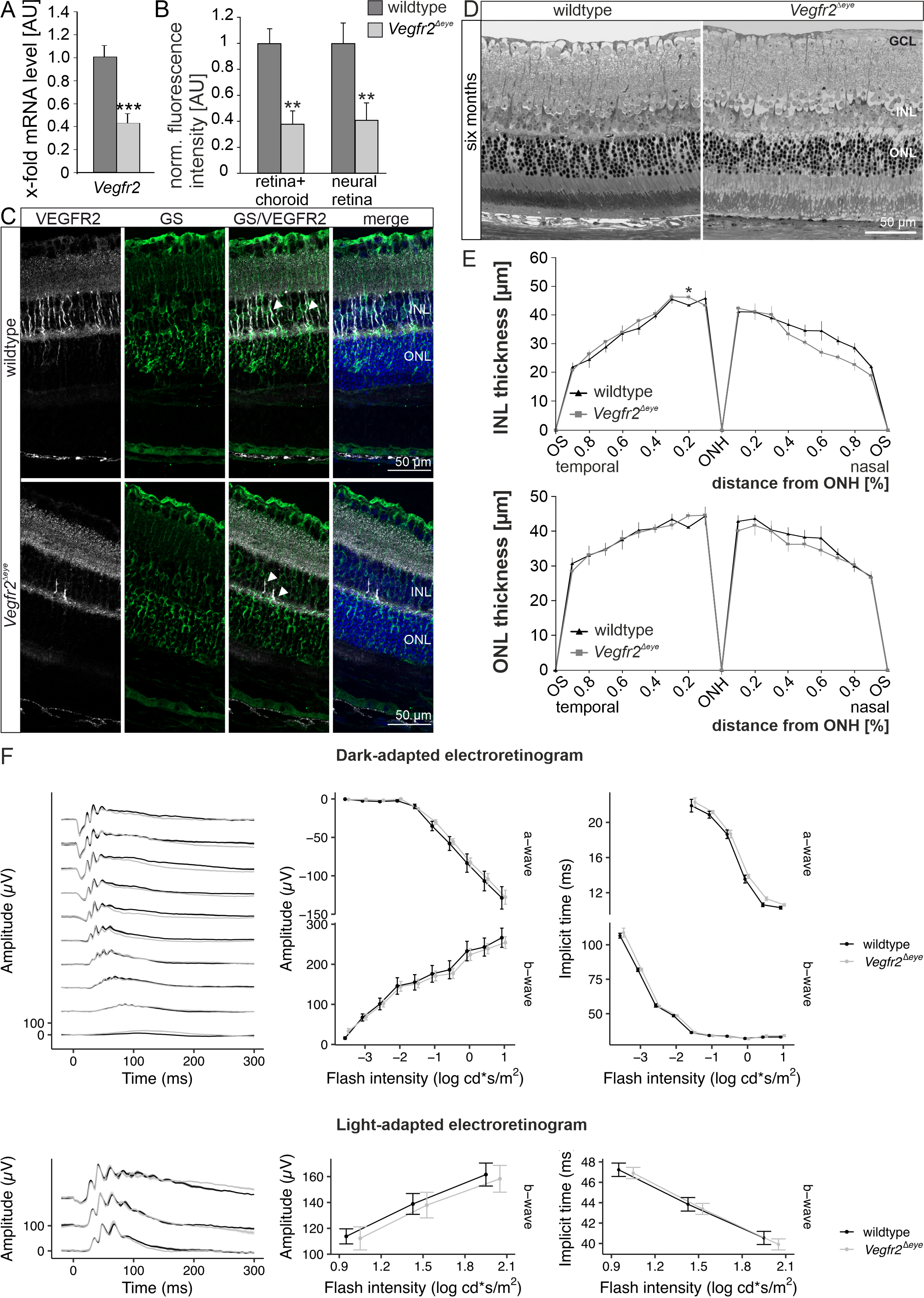


## Supplementary figure 1: Deletion of ocular *Vegfr2* in healthy animals: retinal morphology and function

**A.** QPCR analyses for mRNA of retinal *Vegfr2* in six weeks old *Vegfr2^Δeye^* and wildtype animals (****p* ≤ 0.001, wildtype: n = 7; *Vegfr2^Δeye^*: n= 7, table 4). **B.** Normalized fluorescence intensity of VEGFR2 staining pattern (wildtype: n = 3; *Vegfr2^Δeye^*: n= 3, table 4)**. C.** Immunoreactivity for VEGFR2 (white) and the Müller glia marker glutamine synthetase (GS, green) in the retina of a *Vegfr2^Δeye^* and its wildtype at the age of six weeks. Distinct immunoreactivity for VEGFR2 was detectable in the choroid (arrows) and in the INL with a clear co-localization with GS (arrowheads) in the wildtype retina but was markedly reduced in the *Vegfr2^Δeye^* retina. Nuclei are stained with DAPI (blue). **D.** Richardson-stained, semithin sections of the central retina of six months old *Vegfr2^Δeye^* and wildtype animals**. E.** Spider diagram illustrating the morphometric analyses of the INL and ONL thickness at defined measure points (* *p* = 0.049, wildtype: n = 5; *Vegfr2^Δeye^*: n = 4). **F.** ERG (dark adapted: top row, light adapted: bottom row) waveforms (left) and their averages with SEM to single flashes of increasing intensity showed similar amplitudes (middle) and implicit times (right) of its trough (a-wave) and peak (b-wave) under dark-adapted conditions. Similarly, responses to three single flashes of white light with increasing intensity show similar waveforms and amplitudes of its peaks (b-wave) under light adapted conditions. Wildtype: n = 4; *Vegfr2^Δeye^*: n= 5. GCL = ganglion cell layer; INL = inner nuclear layer; ONL = outer nuclear layer; *Vegfr2* = vascular endothelial growth factor receptor 2. Data are means ± SEM, student`s t-test.


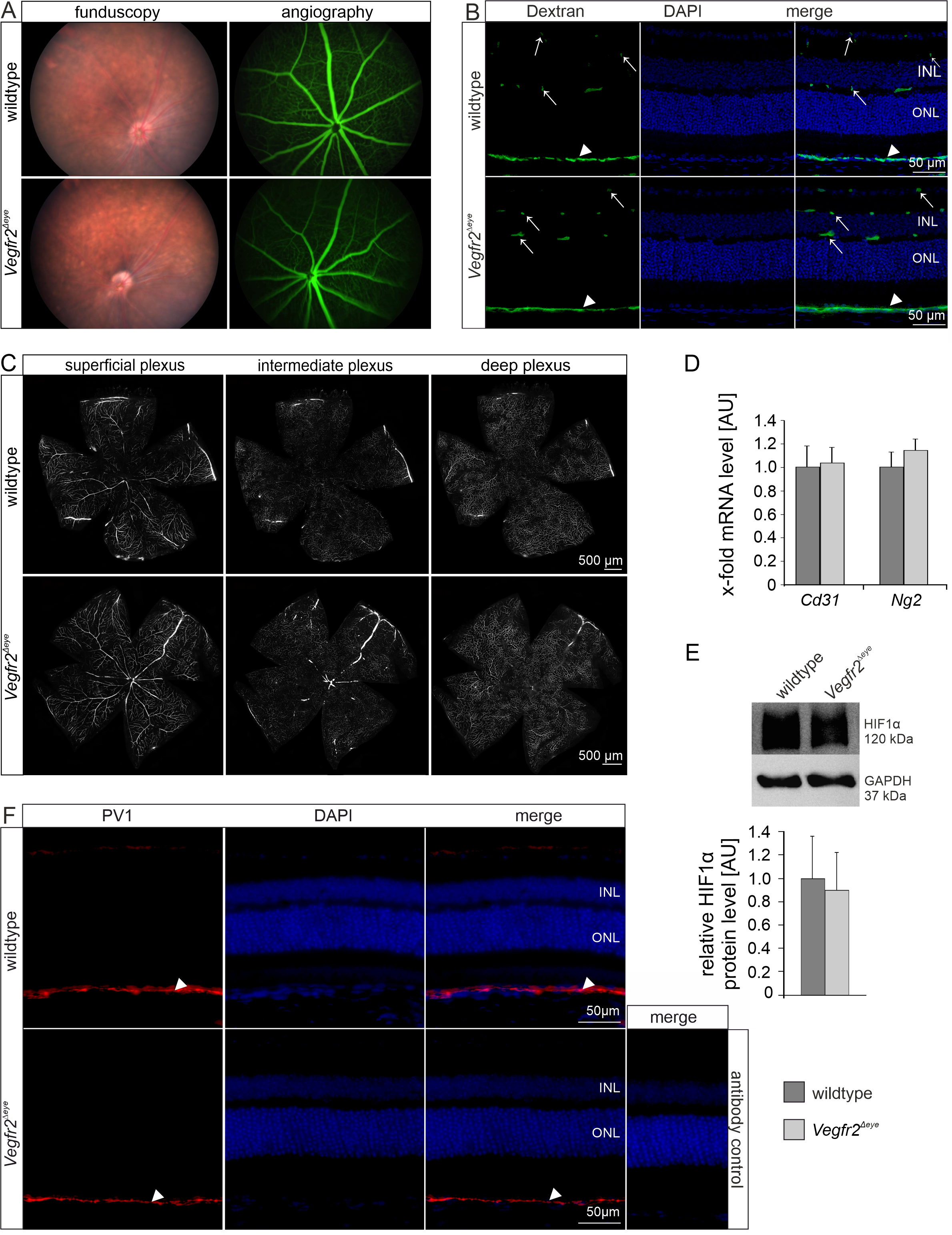


## Supplementary figure 2: Retinal vasculature of *Vegfr2^Δeye^* and wildtype animals

**A.** Funduscopy and fluorescein angiography did not show obvious differences between the three months old *Vegfr2^Δeye^* and wildtype littermate. **B.** FITC-dextran (green, arrows) perfused sections of the posterior part of the eye showed a regular morphology and perfusion of the three intraretinal vascular plexus (arrows) and the choroid (arrowheads) in six weeks old eyes of a *Vegfr2^Δeye^* and wildtype animal. **C.** FITC-dextran perfused retinal flatmounts of a three months old *Vegfr2^Δeye^* and wildtype animal. The three intraretinal plexus (superficial, intermediate and deep) did not show vascular alterations. **D.** QPCR analyses for mRNA of retinal *Cd31 and Ng2* in six weeks old *Vegfr2^Δeye^* and wildtype animals (n ≥ 7, table 4). **E.** Western blot analyses and corresponding densitometric analyses for retinal HIF1α levels in six week old *Vegfr2^Δeye^* and wildtype animals. GAPDH was used as loading control. Wildtype: n = 6; *Vegfr2^Δeye^*: n = 7. **F.** Immunofluorescence staining against PV1 (red, arrowheads) of six weeks old eyes show a regular staining pattern of the fenestrated endothelium of the choriocapillaris. Cell nuclei were DAPI-stained (blue). *Cd31* = cluster of differentiation, *Ng2* = neural/glial antigen 2, HIF1α = hypoxia-inducible factor 1-alpha, PV1 = plasmalemmal vesicle associated protein, Data are means ± SEM. student`s t-test.


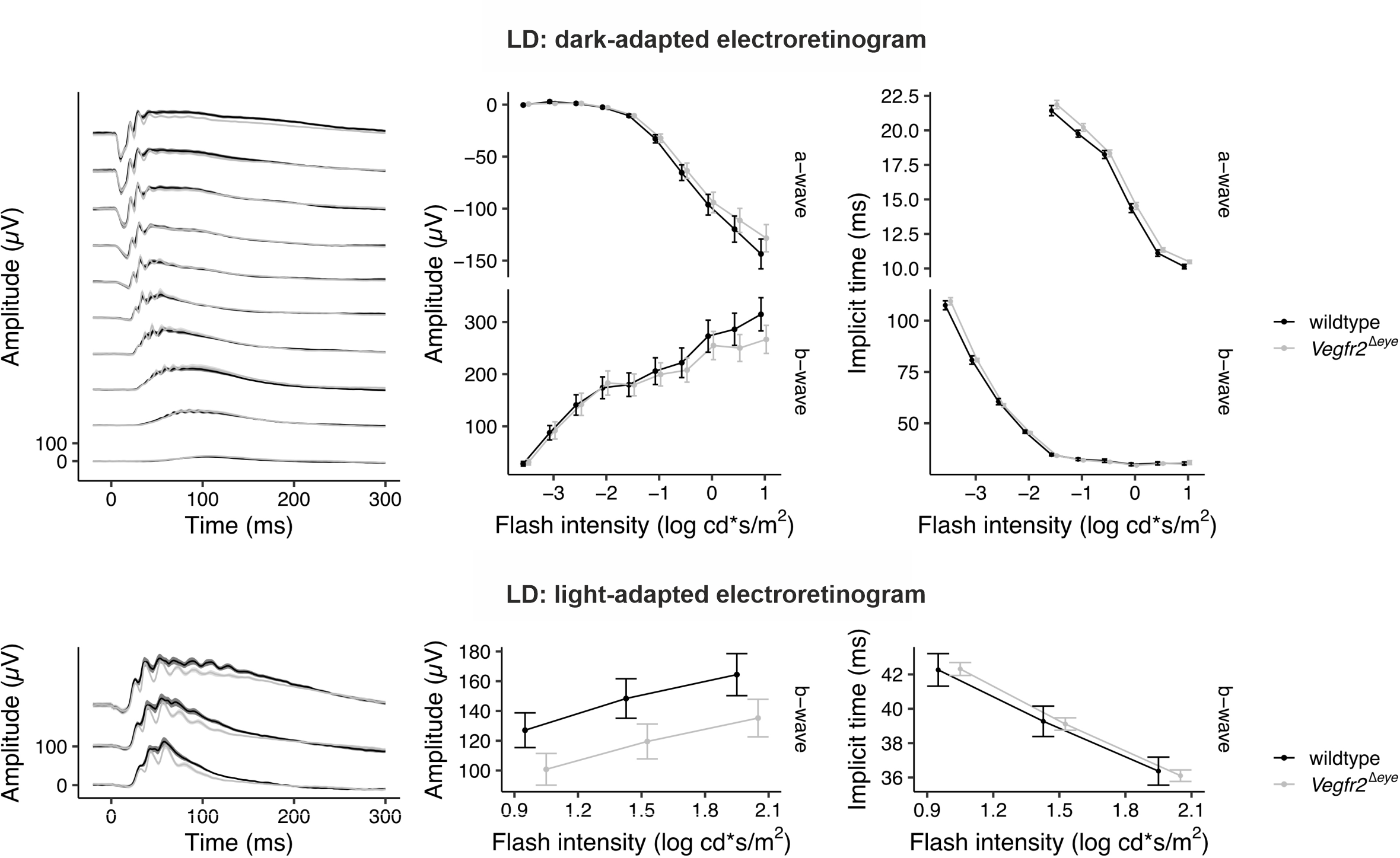


## Supplementary figure 3: ERG of light-damaged animals

ERG (dark-adapted conditions: top row, light-adapted conditions: bottom row) waveforms (left) and their averages with SEM to single flashes of increasing intensity showed slightly reduced amplitudes (middle) of its trough (a-wave) and peak (b-wave) in LD *Vegfr2^Δeye^* but similar implicit times (right) in LD *Vegfr2^Δeye^* and LD wildtypes. Yet, the amplitudes (left) of the responses to single flashes recorded under light-adapted conditions were slightly reduced (middle) while implicit times were similar (right) in LD *Vegfr2^Δeye^* compared to LD wildtypes. Wildtype: n = 8; *Vegfr2^Δeye^*: n= 9. Data are means ± SEM. LD = light-damaged.


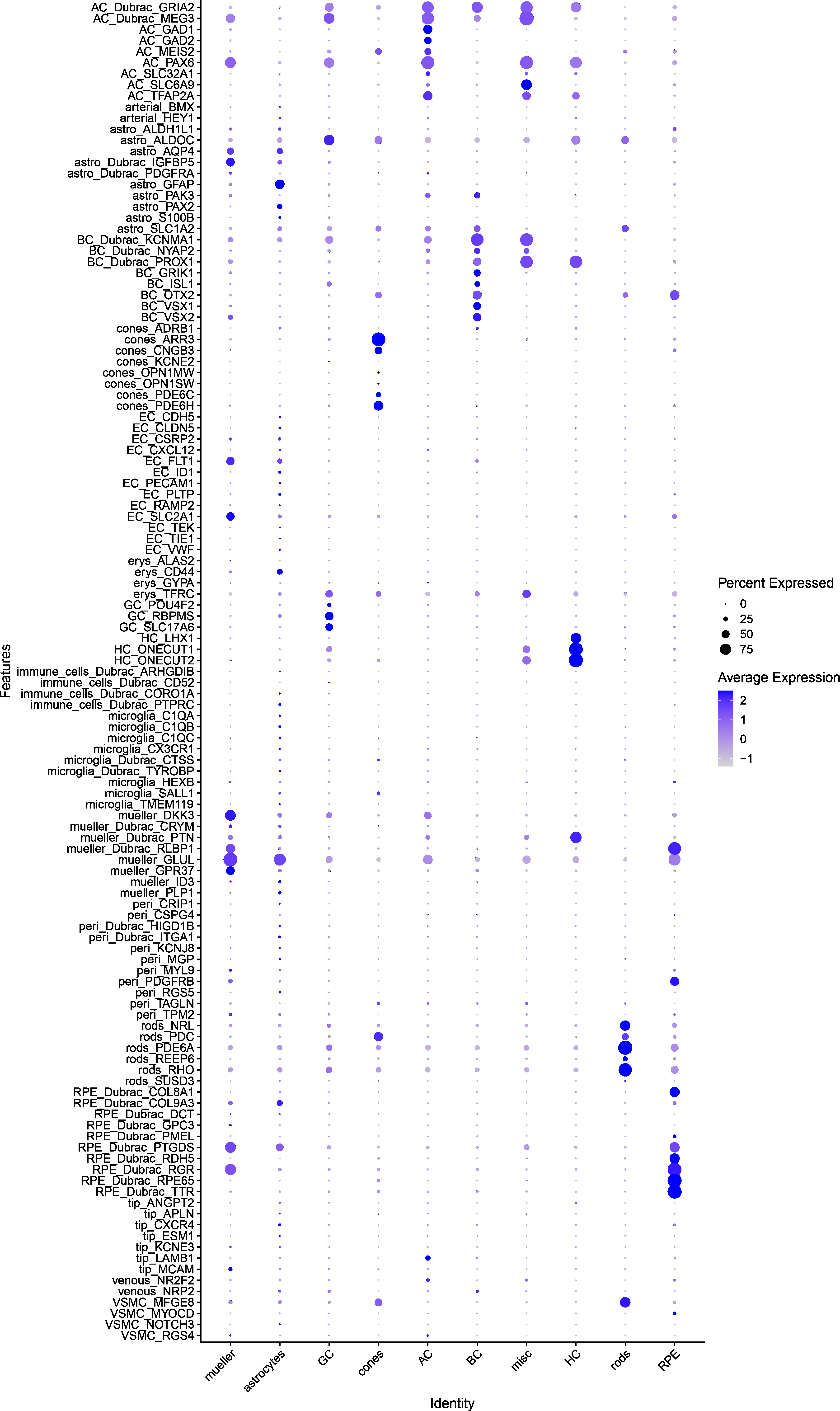


## Supplementary figure 4: Identification of cell types in the human retina following snRNAsequencing

Expression of cell-type markers (columns) vs. identified clusters (rows) as shown in main figure 5 A and B. The percentage of cells in each cluster is represented by the size of the circle and average expression level by color depth.


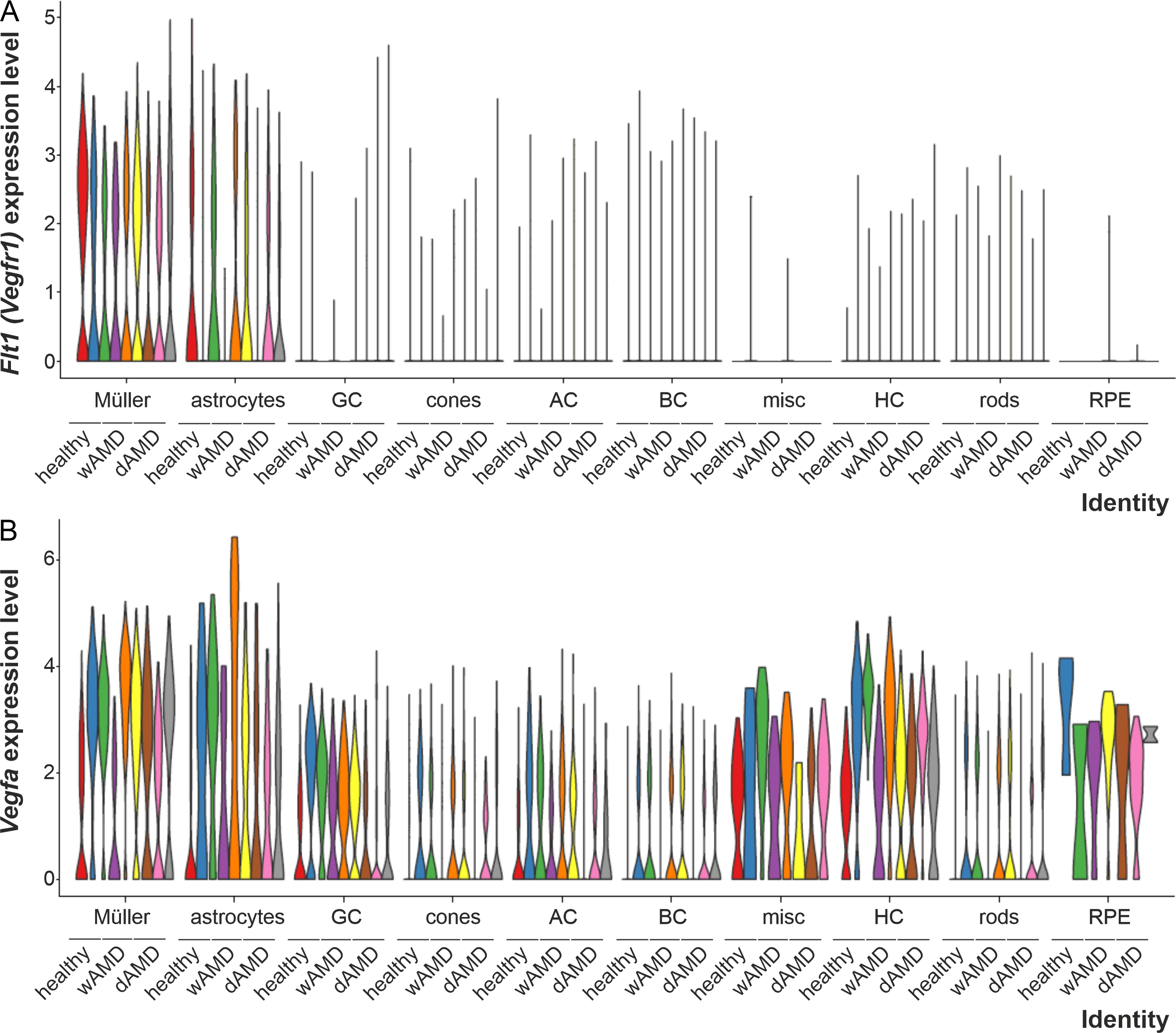


## Supplementary figure 5: Expression of *Vegfa* and *Vegfr1* in single nuclei sequencing data of human healthy retinae, wet AMD and dry AMD.

Violin blots reflecting the expression levels of *Vegfa* (**A**) and *Flt1 (Vegfr1)* (**B**) in the identified retinal cell populations and under healthy, wet AMD and dry AMD conditions are shown. Healthy human donors n = 3, wAMD n = 3, dAMD n = 3. Müller = Müller cells, GC= ganglion cells, AC= amacrine cells, BC= bipolar cells, HC= horizontal cells, RPE= retinal pigment epithelium. *Vegfa* = vascular endothelial growth factor a, *Flt1* = Fms Related Receptor Tyrosine Kinase 1, *Vegfr1/2* = vascular endothelial receptor 1/2, wAMD = wet AMD, dAMD = dry AMD.
