# Supplementary figures and images for: VEGFR2 deletion increases susceptibility to photoreceptor degeneration through glial-neuronal interaction
Source: Cell Death Dis. 2026 Jun 11;17(1):564. doi: 10.1038/s41419-026-08963-z (PMC13260824; doi:10.1038/s41419-026-08963-z)

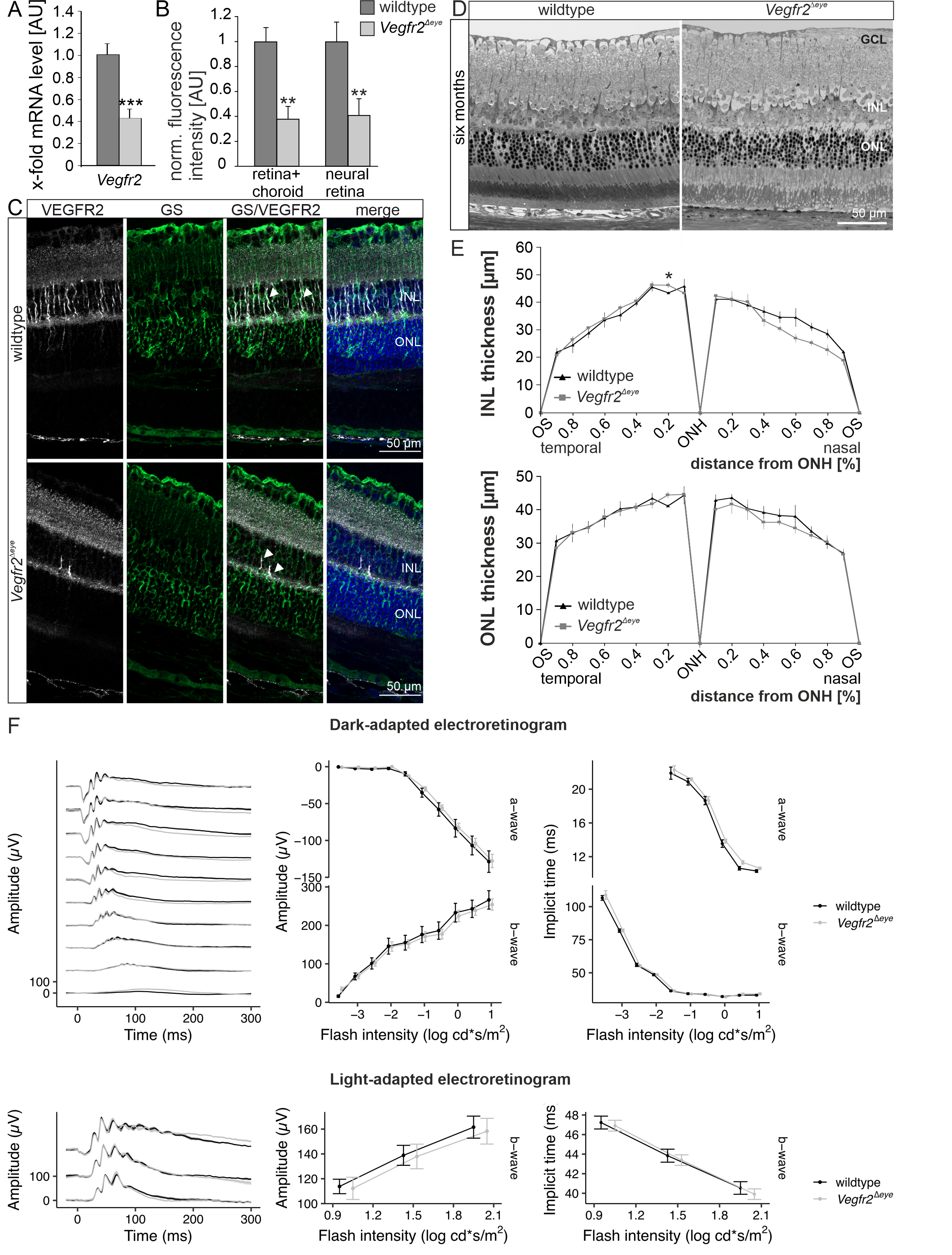

Supplement: Supplementary file 2 — Supplementary figure 1: Deletion of ocular Vegfr2 in healthy animals: retinal morphology and function [file 41419_2026_8963_MOESM2_ESM.tif]

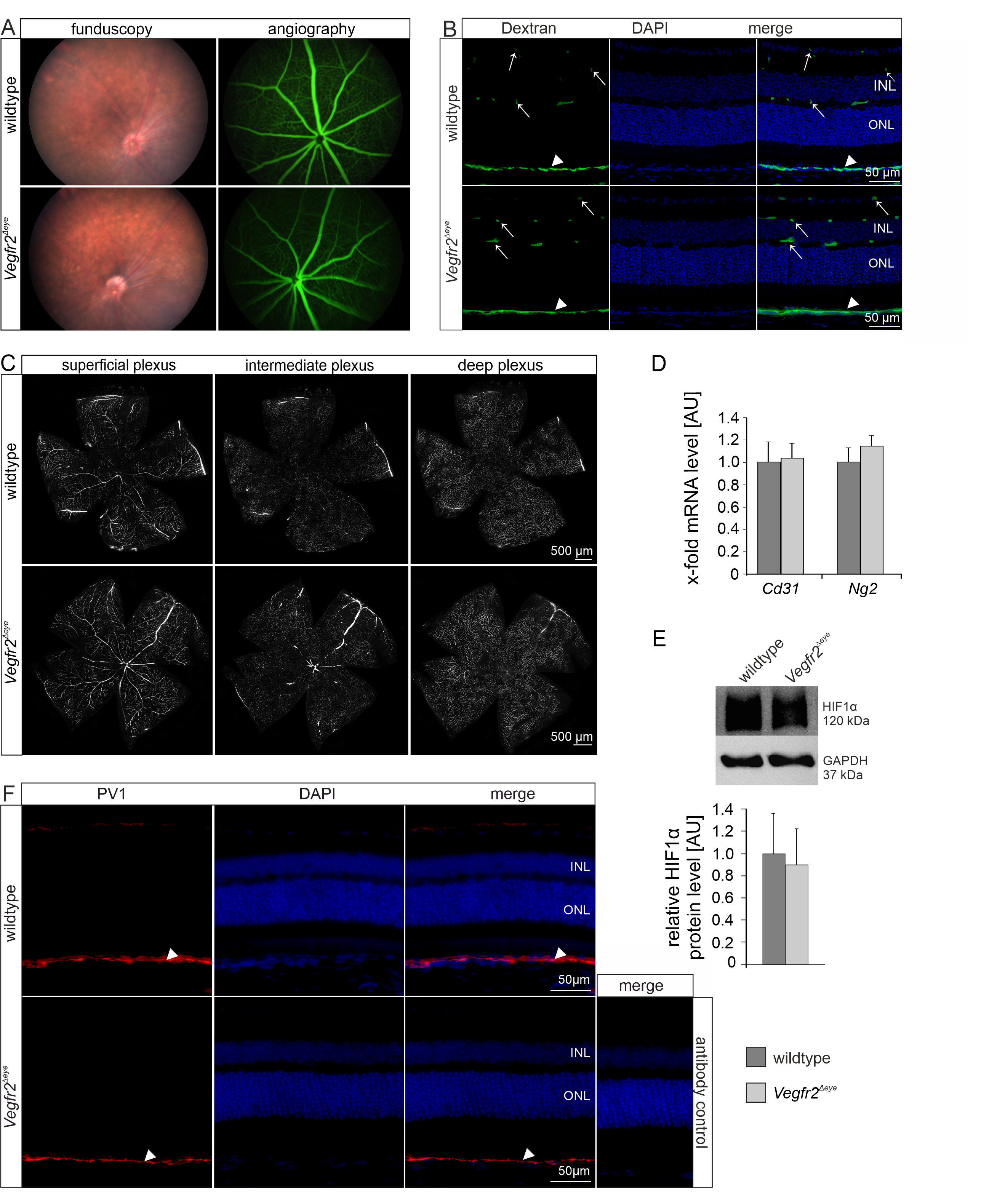

Supplement: Supplementary file 3 — Supplementary figure 2: Retinal vasculature of Vegfr2Δeye and wild-type animals [file 41419_2026_8963_MOESM3_ESM.tif]

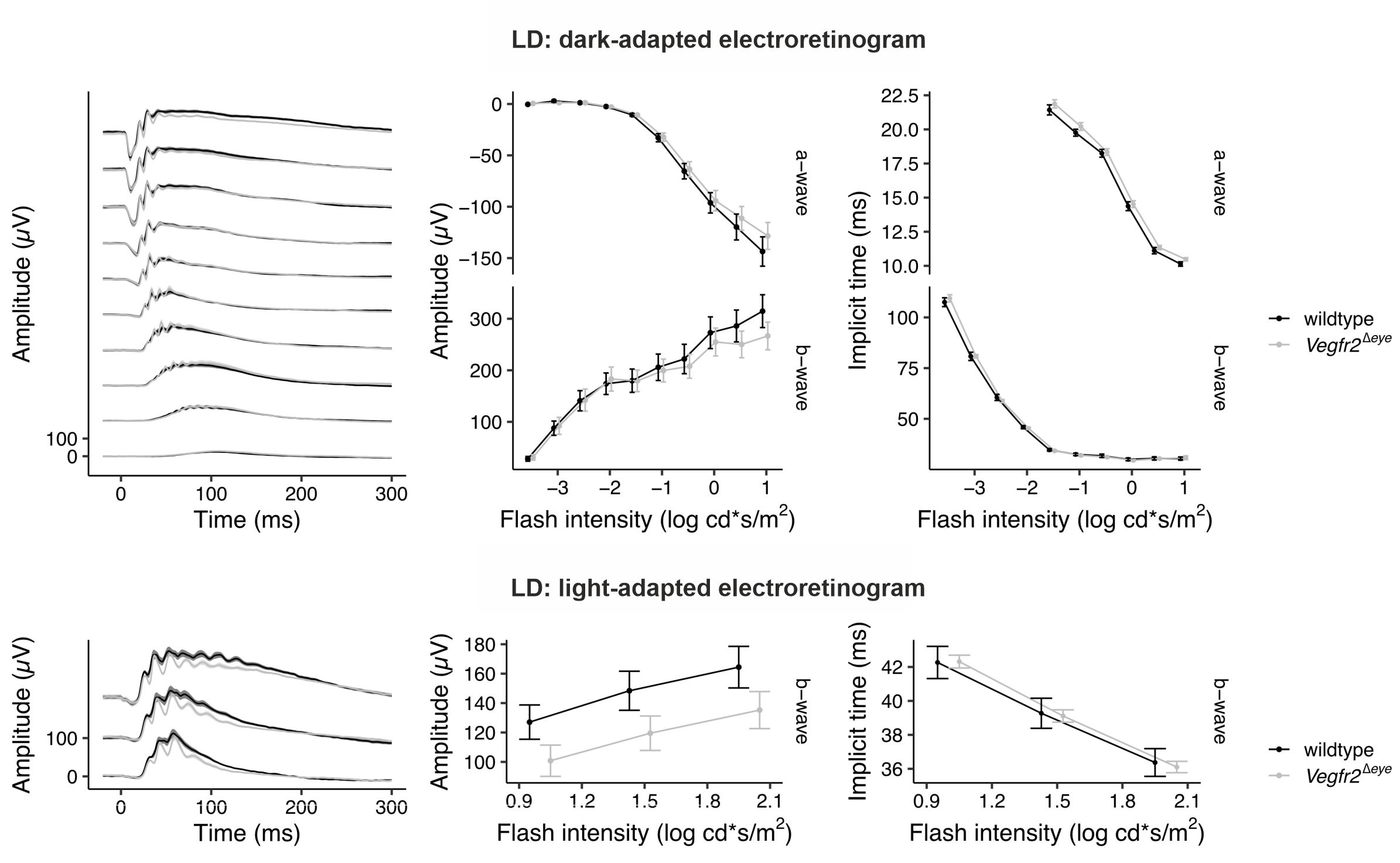

Supplement: Supplementary file 4 — Supplementary figure 3: ERG of light-damaged animals [file 41419_2026_8963_MOESM4_ESM.tif]

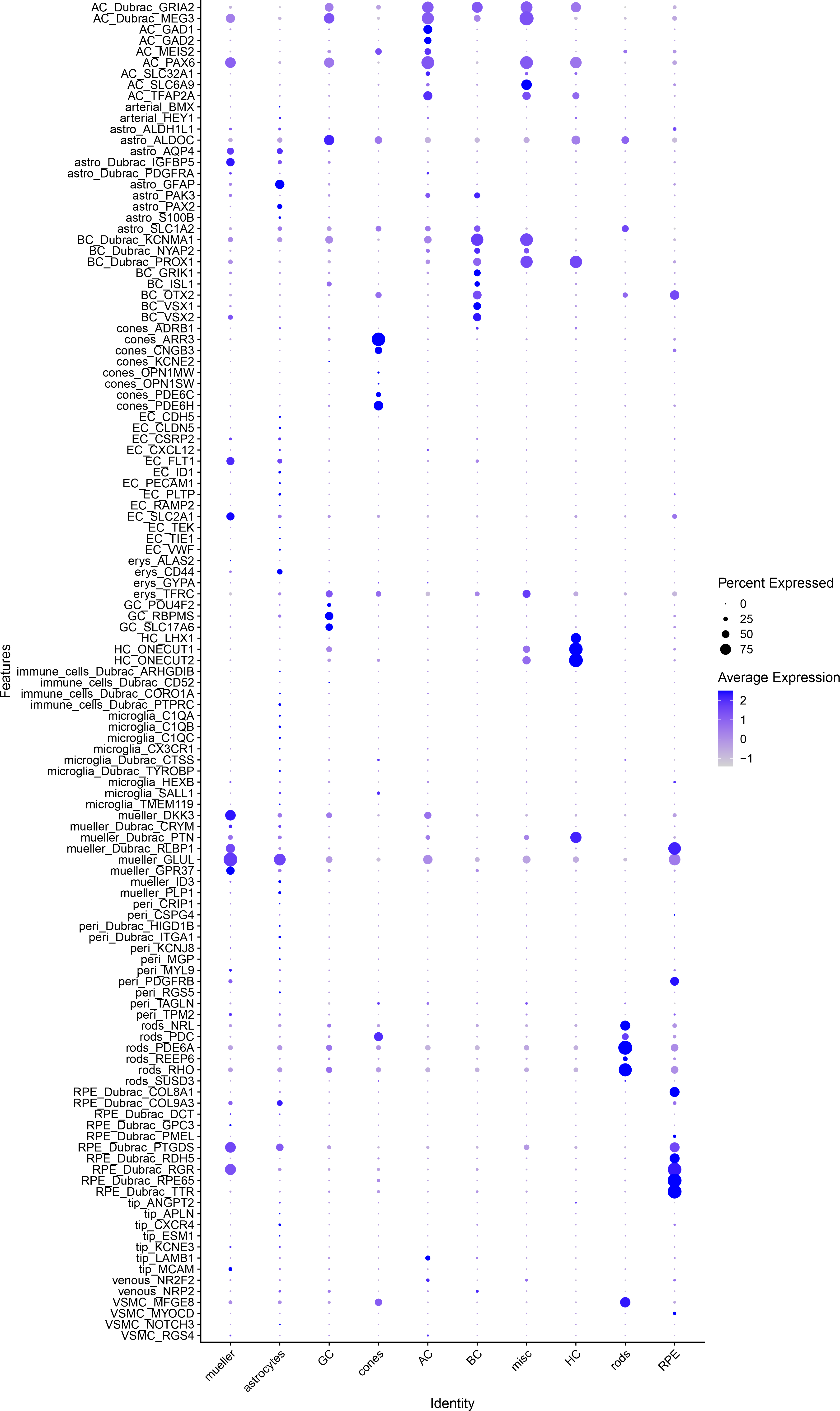

Supplement: Supplementary file 5 — Supplementary figure 4: Identification of cell types in the human retina following snRNA sequencing [file 41419_2026_8963_MOESM5_ESM.tif]

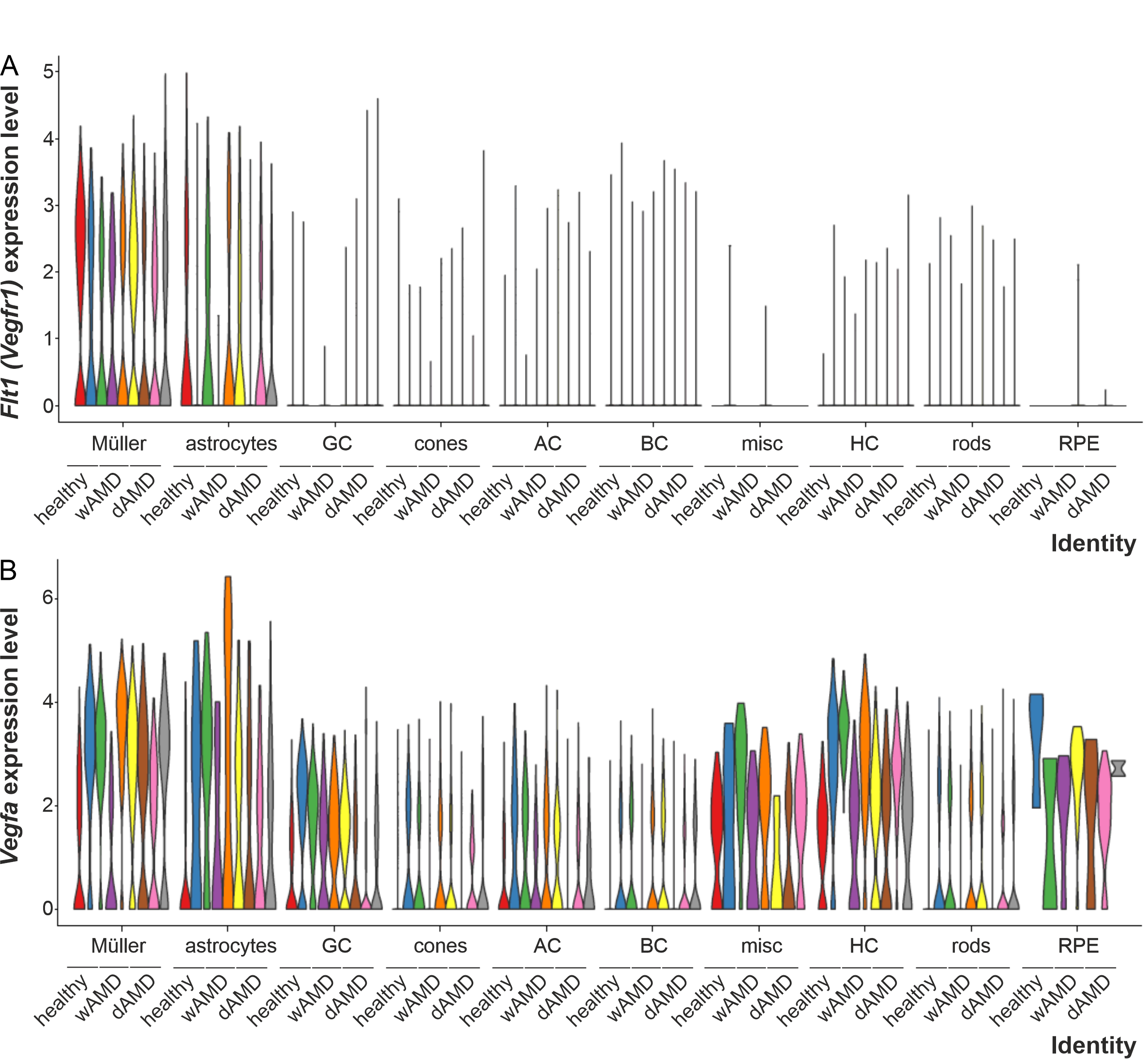

Supplement: Supplementary file 6 — Supplementary figure 5: Expression of Vegfa and Vegfr1 in single nuclei sequencing data of human healthy retinae, wet AMD and dry AMD. [file 41419_2026_8963_MOESM6_ESM.tif]
