# Supplementary material for: VEGFR2 deletion increases susceptibility to photoreceptor degeneration through glial-neuronal interaction
Source: Cell Death Dis. 2026 Jun 11;17(1):564. doi: 10.1038/s41419-026-08963-z (PMC13260824; doi:10.1038/s41419-026-08963-z)
Supplement: Supplementary file 7 — Unedited western blot gels [file 41419_2026_8963_MOESM7_ESM.pdf]

Full and uncropped western blots

Main figure 4C:

pAKT

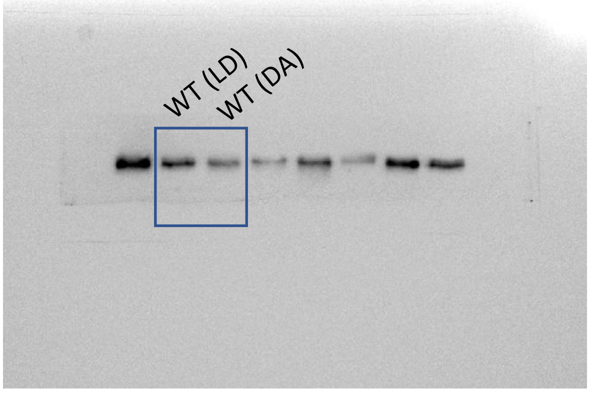

AKT

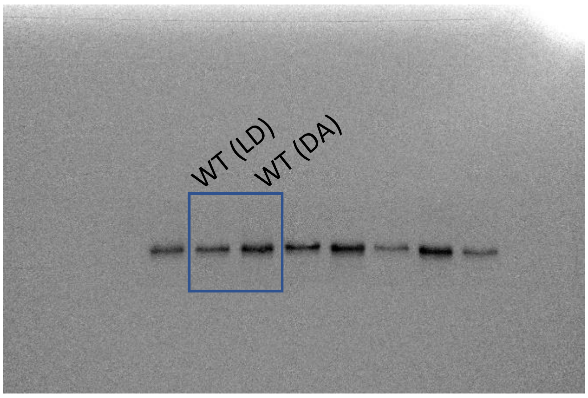

GAPDH

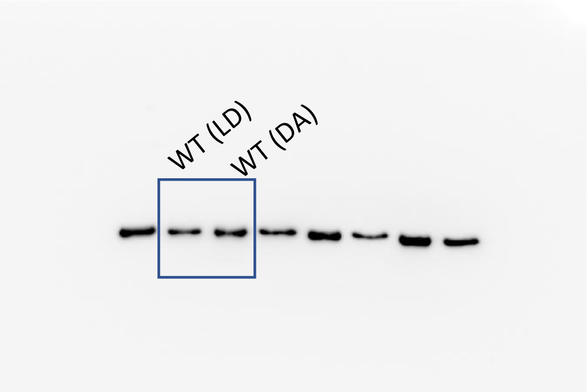

Western blot analyses for retinal AKT and phosphorylated (p) AKT in six weeks old wildtypes: dark-adapted (DA) and 6h following light exposure (LD). The reference protein glyceraldehyde 3-phosphate dehydrogenase (GAPDH) was used as loading control. wildtype = WT.

Main figure 4D:

pAKT:

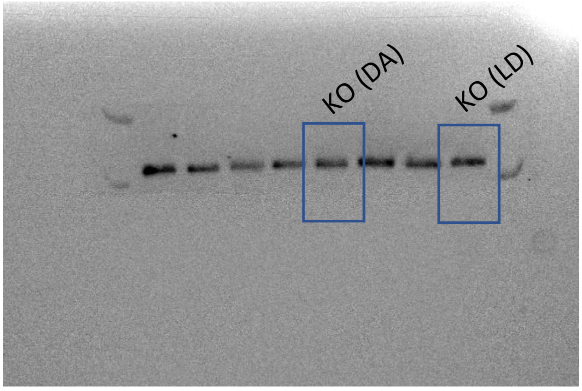

AKT:

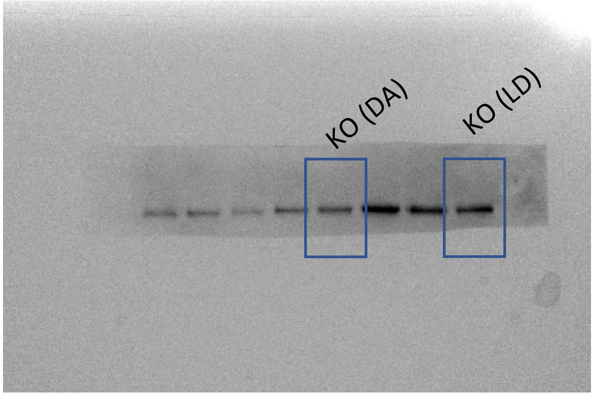

GAPDH:

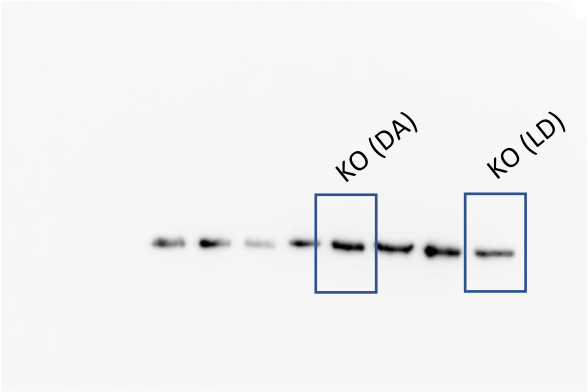

Western blot analyses for retinal AKT and phosphorylated (p) AKT in six weeks old *Vegfr2*$^{\Delta eye}$: dark-adapted (DA) and 6h following light exposure (LD). The reference protein glyceraldehyde 3-phosphate dehydrogenase (GAPDH) was used as loading control. *Vegfr2*$^{\Delta eye}$ = KO

Supplementary figure 2E:

HIF1α:

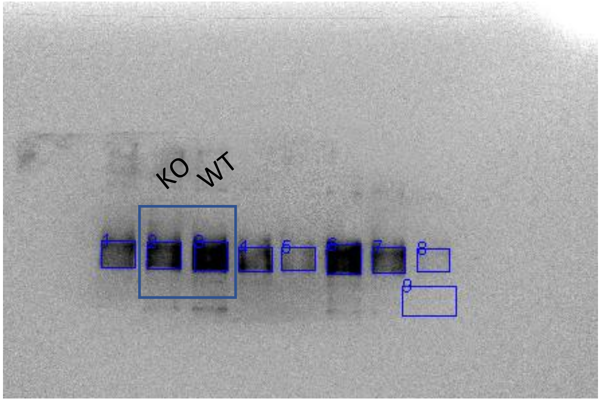

GAPDH:

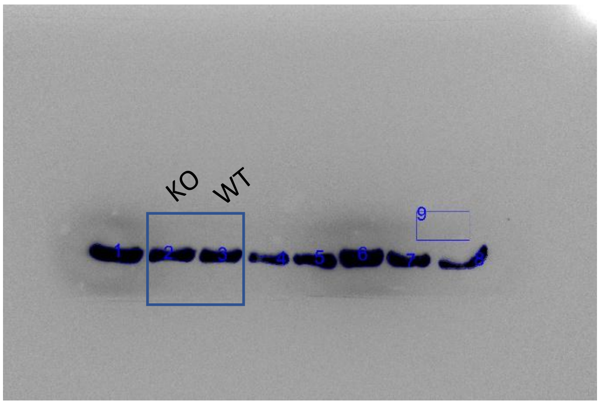

Western blot analyses for retinal HIF1α levels in six week old *Vegfr2*$^{\Delta eye}$ and wildtype animals. Glyceraldehyde 3-phosphate dehydrogenase (GAPDH) was used as loading control.
